# Genome-wide identification and functional characterization of cotton (*Gossypium hirsutum*) MAPKKK gene family in response to drought stress

**DOI:** 10.1186/s12870-020-02431-2

**Published:** 2020-05-14

**Authors:** Jing-Bo Zhang, Xin-Peng Wang, Ya-Chao Wang, Yi-Hao Chen, Jing-Wen Luo, Deng-Di Li, Xue-Bao Li

**Affiliations:** Hubei Key Laboratory of Genetic Regulation and Integrative Biology, School of Life Sciences, Central China Normal University, Wuhan, 430079 China

**Keywords:** Cotton (*Gossypium hirsutum*), Mitogen-activated protein kinase kinase kinase (MAPKKK), Cis-elements, Drought stress, Virus-induced gene silencing (VIGS)

## Abstract

**Background:**

Mitogen-activated protein kinase kinase kinases (MAPKKKs) are significant components in the MAPK signal pathway and play essential roles in regulating plants against drought stress. To explore *MAPKKK* gene family functioning in cotton response and resistance to drought stress, we conducted a systematic analysis of *GhMAPKKKs*.

**Results:**

In this study, 157 nonredundant *GhMAPKKKs* (including 87 *RAFs*, 46 *MEKKs* and 24 *ZIKs*) were identified in cotton (*Gossypium hirsutum*). These *GhMAPKKK* genes are unevenly distributed on 26 chromosomes, and segmental duplication is the major way for the enlargement of MAPKKK family. Furthermore, members within the same subfamily share a similar gene structure and motif composition. A lot of cis-elements relevant to plant growth and response to stresses are distributed in promoter regions of *GhMAPKKKs*. Additionally, these *GhMAPKKKs* show differential expression patterns in cotton tissues. The transcription levels of most genes were markedly altered in cotton under heat, cold and PEG treatments, while the expressions of some *GhMAPKKKs* were induced in cotton under drought stress. Among these drought-induced genes, we selected *GhRAF4* and *GhMEKK12* for further functional characterization by virus-induced gene silencing (VIGS) method. The experimental results indicated that the gene-silenced cotton displayed decreased tolerance to drought stress. Malondialdehyde (MDA) content was higher, but proline accumulation, relative leaf water content and activities of superoxide dismutase (SOD) and peroxidase (POD) were lower in the gene-silenced cotton, compared with those in the controls, under drought stress.

**Conclusion:**

Collectively, a systematic survey of gene structure, chromosomal location, motif composition and evolutionary relationship of MAPKKKs were performed in upland cotton (*Gossypium hirsutum*). The following expression and functional study showed that some of them take important parts in cotton drought tolerance. Thus, the data presented here may provide a foundation for further investigating the roles of *GhMAPKKKs* in cotton response and resistance to drought stress.

## Background

Cotton is an important crop that produces excellent natural fibers extensively used in textile industry around the world [1]. Hence, cotton has been cultivated on a large scale worldwide. Upland cotton (*Gossypium hirsutum*) is an allotetraploid species (AD1, 2n = 4X = 52), and contributes about 90% yield of cotton fibers in the world [2]. According to reports, 57% of cotton worldwide is cultivated in water-deficit areas (World Resources Institute, http://www.wri.org/). Drought stress severely affected cotton production and quality [3]. Therefore, it is important to improve the water-use efficiency of cotton and to generate drought-tolerant cultivars by genetic engineering.

Mitogen-activated protein kinase (MAPK) signal cascades that are highly conserved in eukaryotes are widely involved in plant growth and defense against abiotic stresses [4, 5]. Plants sense environmental stimuli and transmit extracellular signals into intracellular through typical mechanisms and cause a series of responses [6]. MAPK signal cascade acts as a comprehensive signal transduction module to activate plant response to environmental stimuli. First, upstream signals activate mitogen-activated protein kinase kinase kinase (MAPKKK) by phosphorylating them, and then the phosphorylated MAPKKK further phosphorylates mitogen-activated protein kinase kinase (MAPKK) at Ser and Thr residues. Finally, the activated MAPKK further phosphorylates MAPK at Thr residue. Thus, the signal is gradually amplified and relayed to downstream components. The phosphorylated MAPKs can regulate downstream genes through phosphorylating the related enzymes, transcription factors and other signal components. In plants, MAPK signal cascades have important functions in abiotic stress responses such as drought, high salinity and reactive oxygen species [7]. To date, many MAPK signal cascades have been identified in plants. Arabidopsis MEKK1-MKK4/5-MPK3/6 signal pathway is the first found MAPK cascade in plant, which is related to plant innate immunity [4]. Besides, NPK1-MEK1-Ntf6 cascade participates in plant response to tobacco mosaic virus infection [8]. Under drought stress, cotton *GhMAP3K15* phosphorylates *GhMKK4*, then the phosphorylated *GhMKK4* further phosphorylates *GhMPK6*, and at last the phosphorylated *GhMPK6* phosphorylates *GhWRKY59*. Then, the phosphorylated *GhWRKY59* interacts with the W-box cis-element in the promoter of *GhDREB2* to activate its expression. The above genes/proteins constitute a regulatory module that takes part in cotton drought response [9].

MAPKKKs are significant constituent parts of MAPK signal pathway [10]. It has been reported that a rice Raf MAPKKK gene (*DSM1*) is involved in increasing plant drought tolerance [11]. Overexpression of tobacco *NPK1* in maize strengthens drought resistance [12]. In Arabidopsis, overexpressing *MAPKKK18* enhances drought tolerance of the transgenic plants [13]. These results indicate MAPKKKs play crucial roles in plant response and defense against drought stress.

*MAPKKKs* compose a large gene family in plants. It has been reported that there are 80 putative *MAPKKKs* in Arabidopsis, 78 in rice, 155 in wheat, 74 in maize, 59 in cucumber, and 89 in tomato [14–18]. Up to now, however, the information of MAPKKK family is still unclear in upland cotton. At the same time, the recent release of the completed genome sequence and annotation of the latest version of upland cotton provides us a chance to more accurately identify and characterize MAPKKK gene family in the allotetraploid cotton genome [19]. In this study, we identified 157 putative *GhMAPKKKs* by genome-wide scanning in upland cotton. We analyzed their evolutionary relationship, chromosomal distribution, gene structure and motif composition, and detected the transcriptional levels of these genes under normal condition and different abiotic stresses. Moreover, we further investigated the function of the selected MAPKKKs in cotton defense against drought stress by using virus-induced gene silencing (VIGS) technology.

## Results

### Identification of MAPKKKs in cotton

To identify *MAPKKKs* in cotton, the 80 Arabidopsis MAPKKK protein sequences were used as query to carry out a blast search against cotton (*Gossypium hirsutum*) genome database (https://www.cottongen.org). The protein sequences of putative GhMAPKKKs were subjected to SMART and Pfam tools for testing the presence of kinase domain. Each of the identified 157 GhMAPKKKs contains a conserved kinase domain, and the GhMAPKKK proteins vary from 294 to 1433 amino acids in length. The molecular weight of GhMAPKKKs varies 33.51 to 157.75 KDa and the isoelectric point (pI) of GhMAPKKKs varies from 4.4 to 9.68. Bioinformatics analysis predicted that 25 GhMAPKKK proteins may be located in the chloroplast, 35 proteins in the cytoplasm, 12 proteins in the mitochondria, 82 proteins in the nucleus, 2 proteins in the plastid and 1 protein in the vacuole. All the characteristics and chromosome location of the identified *GhMAPKKKs* are shown in Additional file 1 Table S1.

**Fig. 1:**
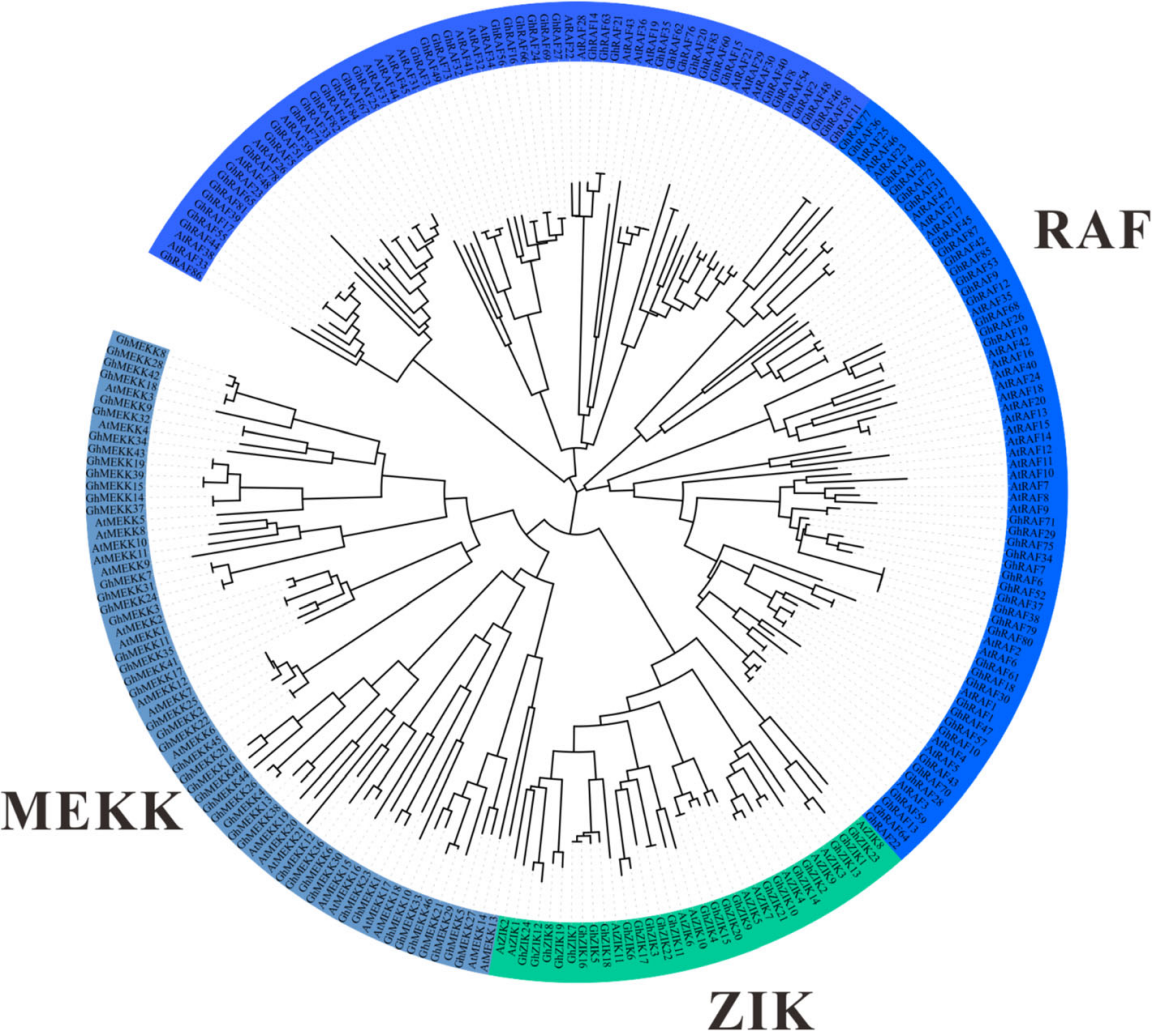
Phylogenetic relationship of GhMAPKKKs with Arabidopsis MAPKKKs. The unrooted phylogenetic tree was constructed using MEGA 6.0 by Neighbor-Joining method and the bootstrap test was performed with 1000 iterations. The three subfamilies are indicated with different colors

We analyzed the evolutionary relationship of *GhMAPKKK*s by constructing a phylogenetic tree according to the multiple sequence alignment of 157 GhMAPKKK protein sequences and 80 Arabidopsis MAPKKK protein sequences. Based on phylogenetic analysis, we found that cotton MAPKKK family can be divided into RAF, MEKK and ZIK groups, similar to those Arabidopsis MAPKKKs (Fig. 1). The RAF group contains 87 members, MEKK group includes 46 members, and ZIK group has 24 members in cotton (Additional file 1 Fig. S1). They were designated as *GhRAF1* – *GhRAF87*, *GhMEKK1* – *GhMEKK45*, and *GhZIK1* – *GhZIK23* in terms of their exact positions on cotton (*G. hirsutum*) chromosomes 1–26 (Additional file 1 Table S1). Additionally, predictive analytics of subcellular localization showed that most cotton RAFs are located in the nucleus and cytoplasm, and ZIKs and MEKKs are often distributed in the nucleus (Additional file 1 Table S1). Furthermore, the *GhMAPKKKs* display an interspersed spread in most clades, indicating the *GhMAPKKK* gene family expanded before the divergence of the lineages. We also observed that a lot of Arabidopsis MAPKKKs have two or more paralogous genes in cotton, indicating that *GhMAPKKKs* are duplicated after divergent evolution of cotton and Arabidopsis.

### Chromosomal location and gene duplication of *GhMAPKKKs*

**Fig. 2:**
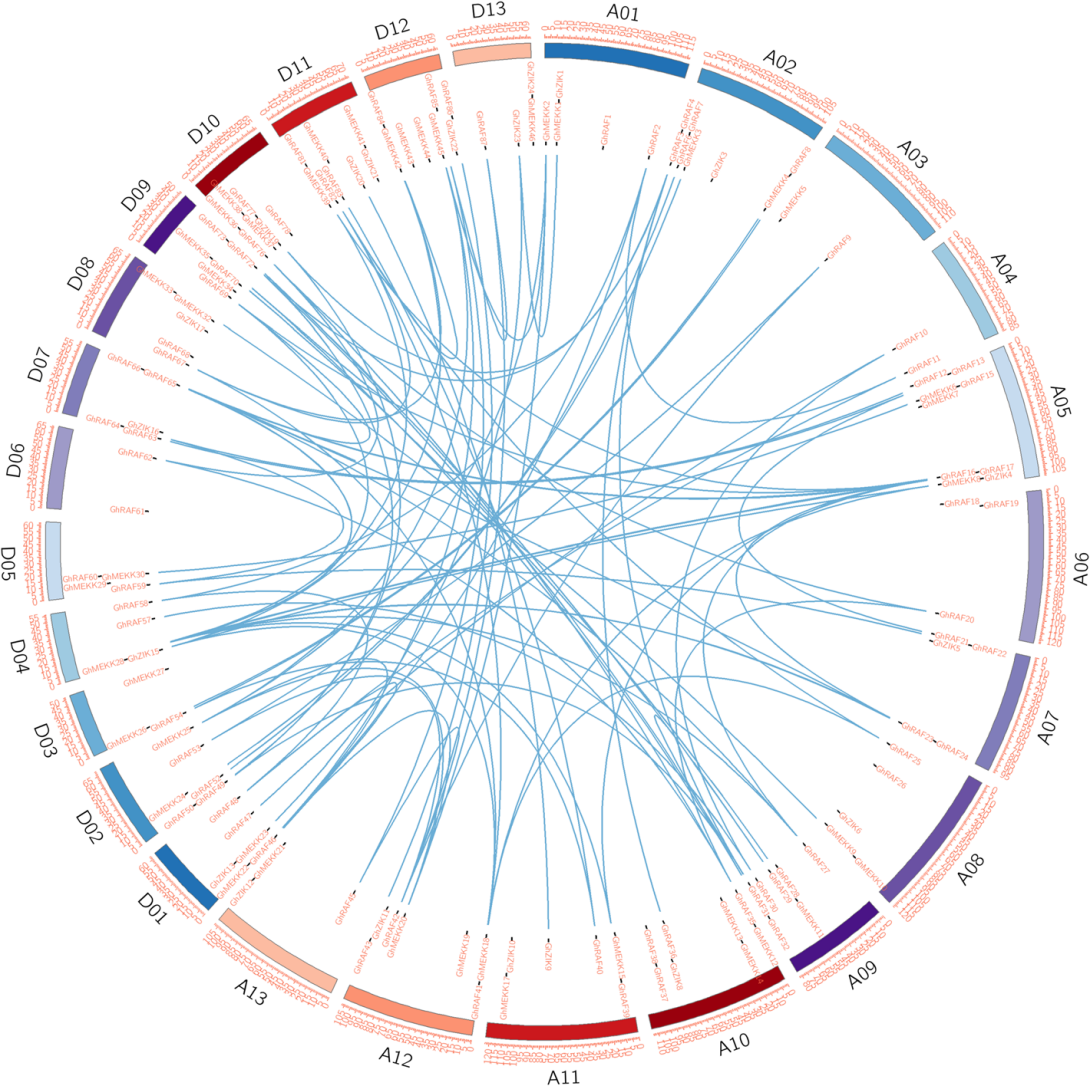
Chromosomal distribution and gene duplication of *MAPKK* genes in cotton (*Gossypium hirsutum*)***.*** The scale is in megabases (Mb), the value on each chromosome represents chromosome length, the gene positions are indicated at each chromosome using black line, and the paralogous *GhMAPKKKs* are connected with a blue line

To determine the location of *GhMAPKKKs* on chromosomes, the information about the chromosomal location was obtained through BLASTN searches from cotton genome database. As shown in Figs. 2, 155 *GhMAPKKKs* were unevenly distributed on different chromosomes, but the remaining two genes were unmapped on chromosomes. Furthermore, cotton genome includes A-subgenome and D-subgenome that contain 80 and 77 *GhMAPKKKs*, respectively, and the duplication events may illuminate the mechanism about the expansion of *GhMAPKKK* gene family. Therefore, we detected the gene pairs in *GhMAPKKK* family. A total of 115 gene pairs were detected in *GhMAPKKK* gene family, and some genes repeatedly participate in gene duplication events (Fig. 2). In these gene pairs, 112 pairs are distributed on diverse chromosomes, suggesting that segmental duplication is the primary expansion model of cotton *MAPKKK* gene family. On the contrary, a few paralogous genes are located on the same chromosomes, indicating tandem duplication also contributes to the expansion of GhMAPKKK family.

Upland cotton (*G. hirsutum*) is an allotetraploid species, which is formed by hybridization of diploid A genome species (*G. arboreum*) and D genome species (*G. raimondii*) [20]. Therefore, we investigated the orthologous genes of *GhMAPKKKs* in its ancestral diploid A genome species (*G. arboreum*) and D genome species (*G. raimondii*). We identified 77 and 76 orthologous gene pairs within the At and Dt subgenomes, respectively, of upland cotton and their corresponding genomes of ancestral A and D diploid cotton species (Additional file 1 Table S2). Moreover, we found that *GhRAF37* in A subgenome have no ortholog in *G. arboretum*, and *GhRAF65*, *GhRAF80* and *GhMEKK30* in D subgenome have no orthologs in *G. raimondii*, suggesting that these genes may be generated after a polyploidization event in *Gossypium*.

### Gene structures and conserved protein motifs of cotton MAPKKKs

**Fig. 3:**
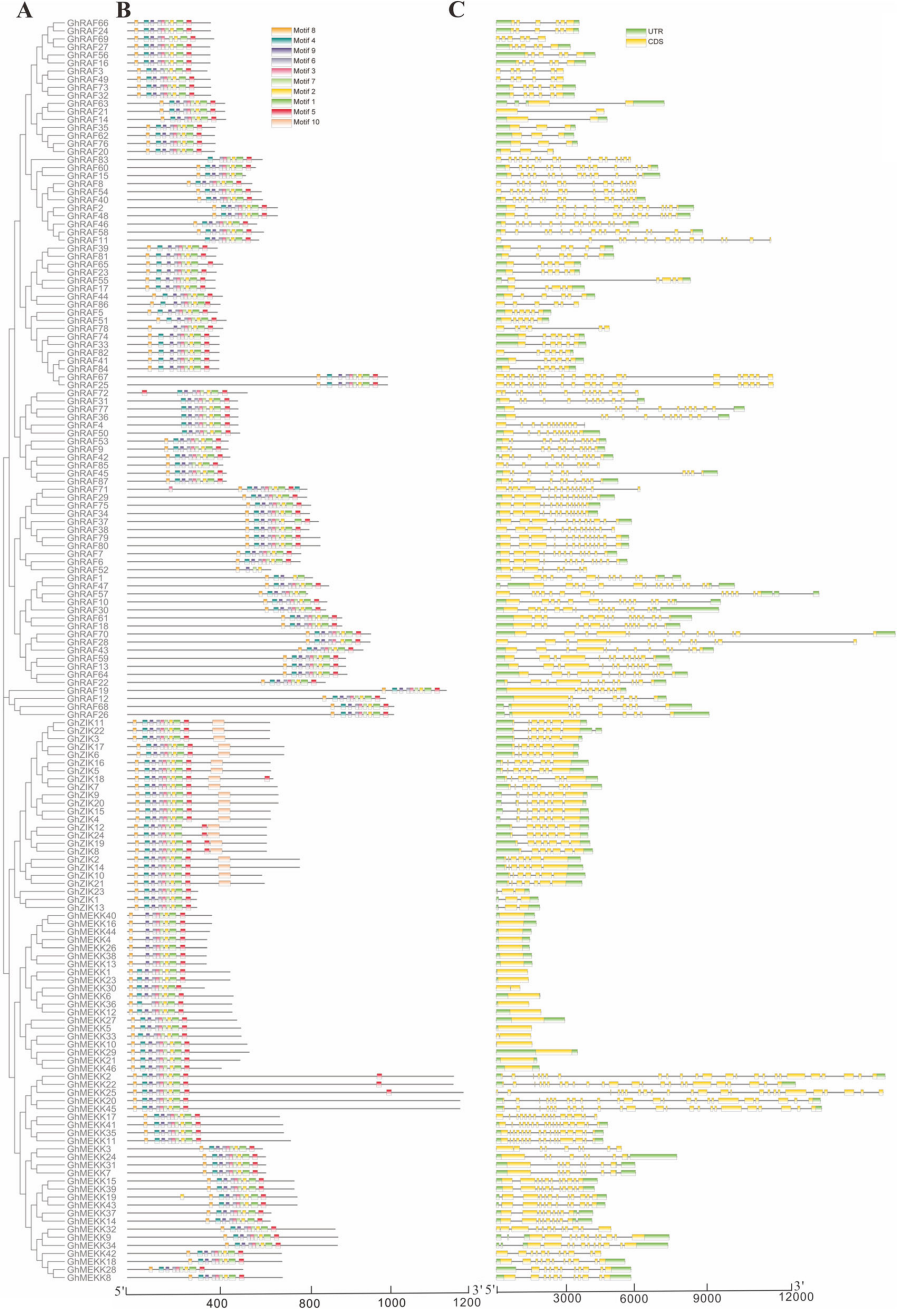
Phylogenetic analysis, conserved motifs and gene structure of MAPKKK family in cotton (*Gossypium hirsutum*). **a** The phylogenetic tree of all MAPKKKs in cotton. **b** The conserved protein motifs were identified in cotton MAPKKK family. Each motif is indicated with a specific color. **c** The exon/intron organization of cotton *MAPKKK* genes. Yellow boxes represent exons and black lines indicate introns

The conserved motif of MAPKKK proteins was investigated using MEME program. As shown in Fig. 3, GhMAPKKK proteins possess 10 motifs. Then these motifs were subjected to SMART online server to annotate, and the results showed that motif 1–9 belong to the Pkinase domain (PF07714). Furthermore, most GhMAPKKK proteins share similar motif composition. On the other hand, we also found some GhMAPKKK proteins have different motif composition. For instance, GhRAF83 lacks motif 8 and 9, while *GhRAF4/31/36/50/77* lack motif 8 in Pkinase domain. Only *GhZIK* proteins have motif 10 which belong to Oxidative-stress-responsive kinase 1 C-terminal domain (PF12202), implying the motif 10 may be related to specific function of the ZIK group.

The exon/intron distribution of *GhMAPKKKs* was investigated by using GSDS tool (http://gsds.cbi.pku.edu.cn/). We found that most *GhMAPKKKs* possess multiple exons and introns, while 19 *GhMEKK* genes contain no intron in their coding regions. To explore if the gene structure of *GhMAPKKKs* is in agreement with the evolutionary relationship, we conducted a phylogenetic analysis of GhMAPKKK protein sequences. As expected, we found there is semblable exon/intron distribution pattern between genes with close evolutionary relationship (Fig. 3).

### Cis-elements existed in promoter regions of *GhMAPKKKs*

We analyzed promoter regions of *GhMAPKKKs* to understand their roles in more depth. The 2 kb sequences upstream from the transcription start site were extracted from cotton genome database and were submitted to PLACE database to investigate their cis-elements distribution. Many cis-elements involved in plant development and in response to environmental stresses were observed in the *GhMAPKKK* promoters (Additional file 1 Table. S3). Out of the 157 *GhMAPKKKs*, 97 have ABREs (ABA-responsive elements), and 22 contain DREs (dehydration responsive elements) in their promoter regions. Each of these 97 genes has an average of 4 ABREs, while each of the 22 genes only contains one DRE in the promoter region (Additional file 1 Fig. S2). The above results imply that *GhMAPKKK*s may play significant roles in plant response to drought stress.

### Expression patterns of *GhMAPKKK*s in cotton tissues

**Fig. 4:**
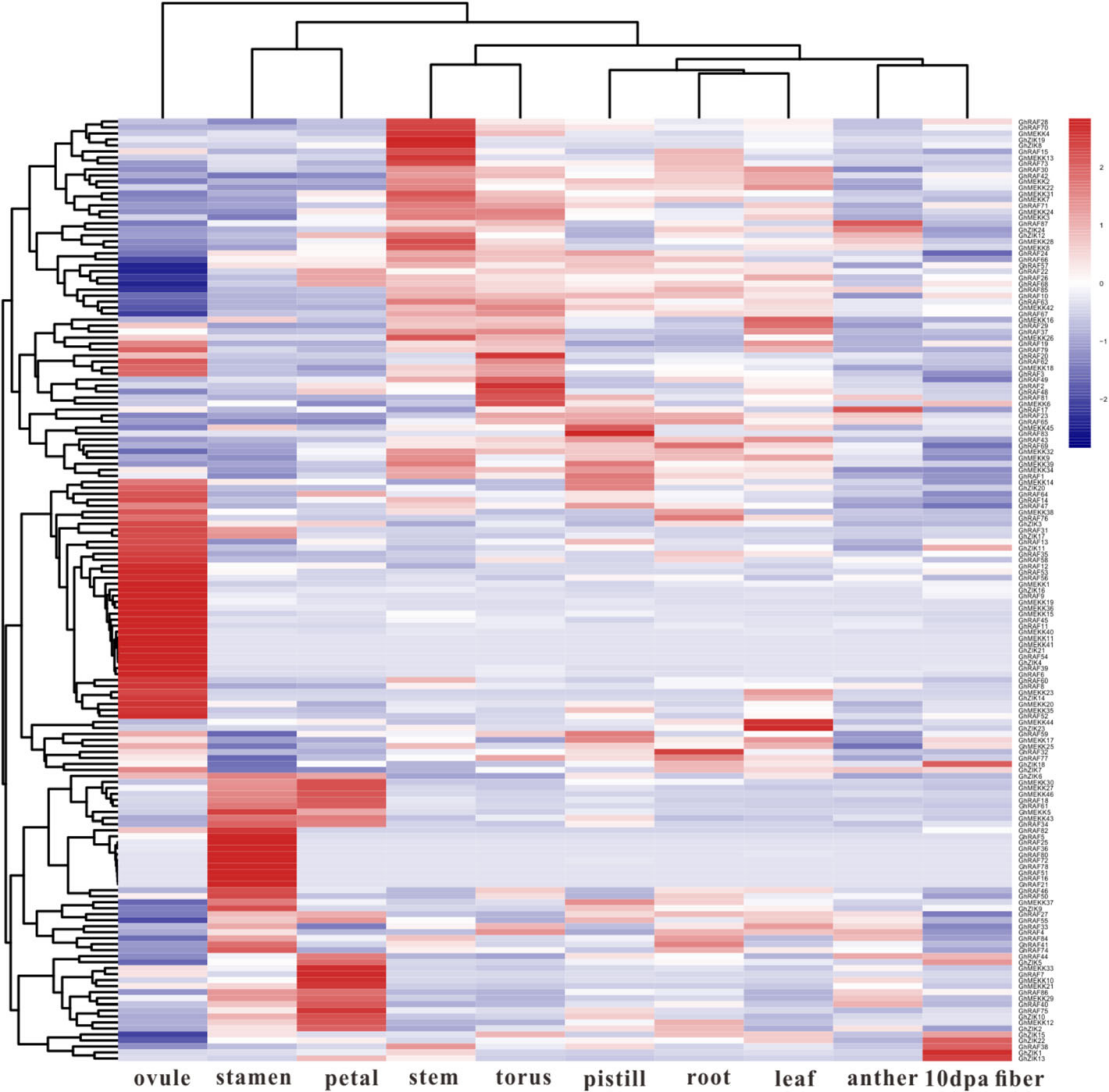
Expression patterns of *GhMAPKKKs* in cotton tissues. The transcriptomic data related to tissue expression were accessed from NCBI and the pheatmap package was used for the generation of heatmaps

To further understand the functions of *GhMAPKKKs*, their transcription patterns in cotton tissues were determined by analyzing cotton transcriptome data. As shown in Fig. 4, *GhMAPKKKs* display different expression patterns among various organs and tissues. Furthermore, some *GhMAPKKKs* are highly expressed in a specific tissue of cotton. For instance, the transcripts of *GhRAF5/25/36/72/78/81* are accumulated mainly in stamen, suggesting these genes may be necessary for stamen growth and development. On the other hand, some *GhMAPKKKs* have higher accumulation of transcripts in some vegetative organs. For instance, higher expression levels of *GhRAF32/78* and *GhZIK7/18* were detected in roots, while *GhMEKK17/25/44* and *GhZIK23* showed strong expression in leaves. The above results suggest *GhMAPKKKs* may participate in cotton growth and developments.

### Expression profiles of *GhMAPKKKs* in cotton under different abiotic stresses

**Fig. 5:**
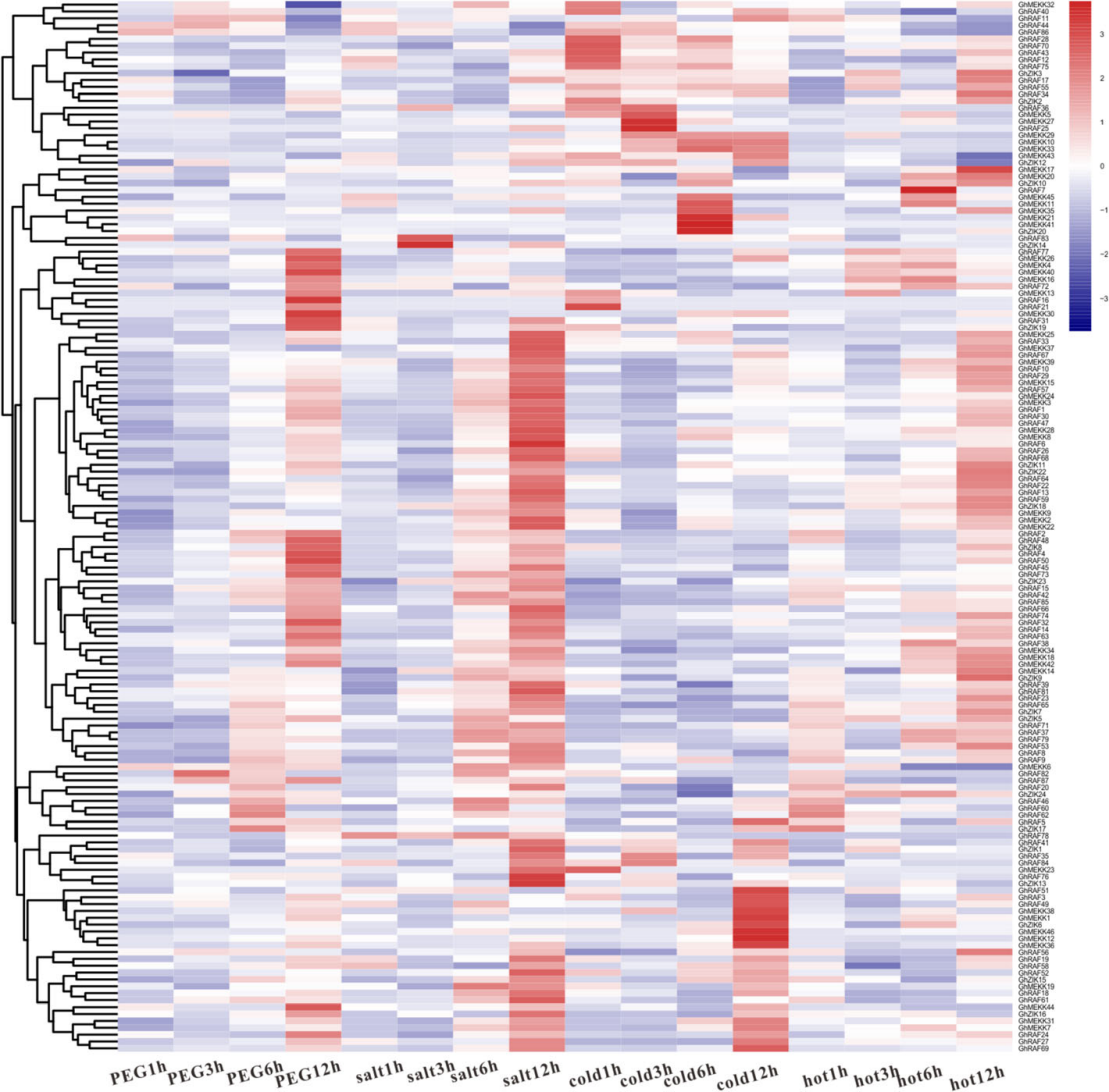
Expression patterns of *GhMAPKKKs* in cotton under different abiotic stresses. Abiotic stress related transcriptomic data were obtained from NCBI, and its normalization and visualization were performed using the pheatmap package

To investigate whether *GhMAPKKKs* are stress-responsive regulatory proteins, we detected the expressions patterns of *GhMAPKKKs* in cotton leaves under various abiotic stresses. As shown in Fig. 5, the expression of most *GhMAPKKKs* was induced in cotton leaves by abiotic stress. Additionally, more *GhMAPKKKs* were significantly induced in leaves under osmotic stress (PEG treatment) and high salinity stress than those under hot and cold stresses. It should be noticed that the expression of osmotic-induced *GhMAPKKKs* are also increased in cotton leaves under high salinity stress upon most occasions. For example, *GhRAF2*, *GhRAF4*, *GhRAF48*, *GhRAF50* and *GhZIK8* are significantly induced in cotton under PEG and salt treatments for 12 h. Additionally, some *GhMAPKKKs* are also induced in cotton under cold and hot treatments. For instance, the expression of *GhRAF3*, *GhRAF49* and *GhMEKK38* is increased in cotton under cold treatment for 12 h, while the transcripts of *GhRAF7* and *GhMEKK45* are increased in cotton under hot treatment for 6 h.

**Fig. 6:**
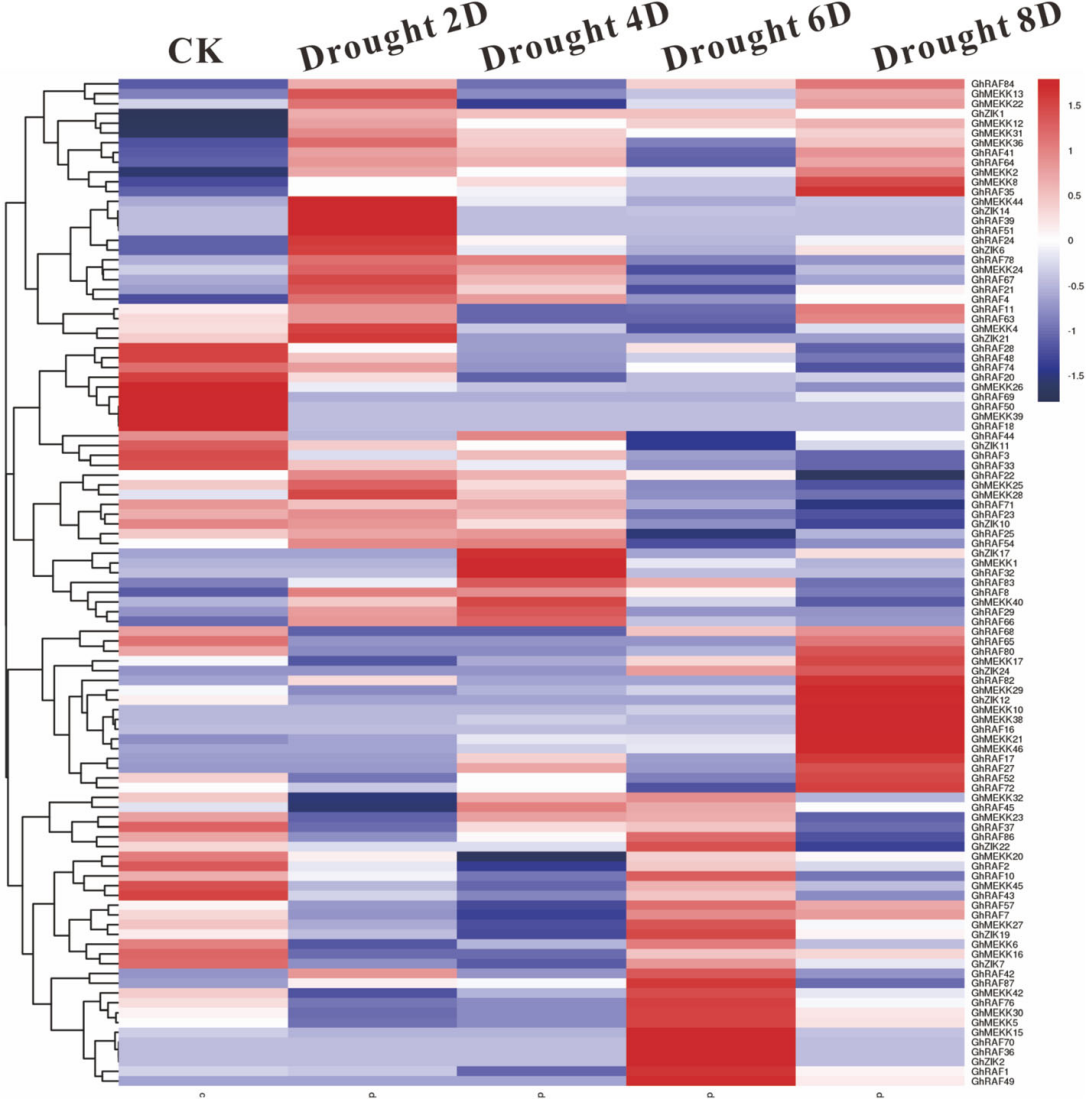
Expression patterns of *GhMAPKKKs* in cotton under drought stress. Drought stress related transcriptomic data were obtained from our lab, and visualization was performed using the pheatmap package

MAPKKKs have been reported to take part in plant resistance to drought. For example, overexpression of tobacco *NPK1* in maize increased the ability of the transgenic plants to defense drought stress [11]. To illuminate the possible functions of *GhMAPKKKs* in cotton resistance to drought, the transcription patterns of *GhMAPKKKs* in cotton under drought stress were determined by analyzing our previous transcriptome data, which was performed with the leaves of a drought-resistant cotton cultivar J-13 under drought treatment for 0, 2, 4, 6 and 8 days, respectively [21]. Expression of most *GhMAPKKKs* was induced in cotton leaves by drought treatment. However, the transcription levels of *GhRAF18*, *GhRAF50*, *GhRAF69* and *GhMEKK39* were decreased in cotton leaves under drought stress. Furthermore, the expression of *GhMEKK12*, *GhMEKK31* and *GhMEKK36* was continuously induced in cotton under drought stress, while the transcriptional levels of some *GhMAPKKKs* were increased just in one or two drought periods (Fig. 6). Furthermore, we found some *GhMAPKKKs* contain DRE elements in their promoter regions (Additional file 1 Figure S2), and paid attention to the expression of these genes. However, only expression of *GhRAF4* and *GhMEKK12* was strongly induced in cotton by drought stress (Fig. 6).

**Fig. 7:**
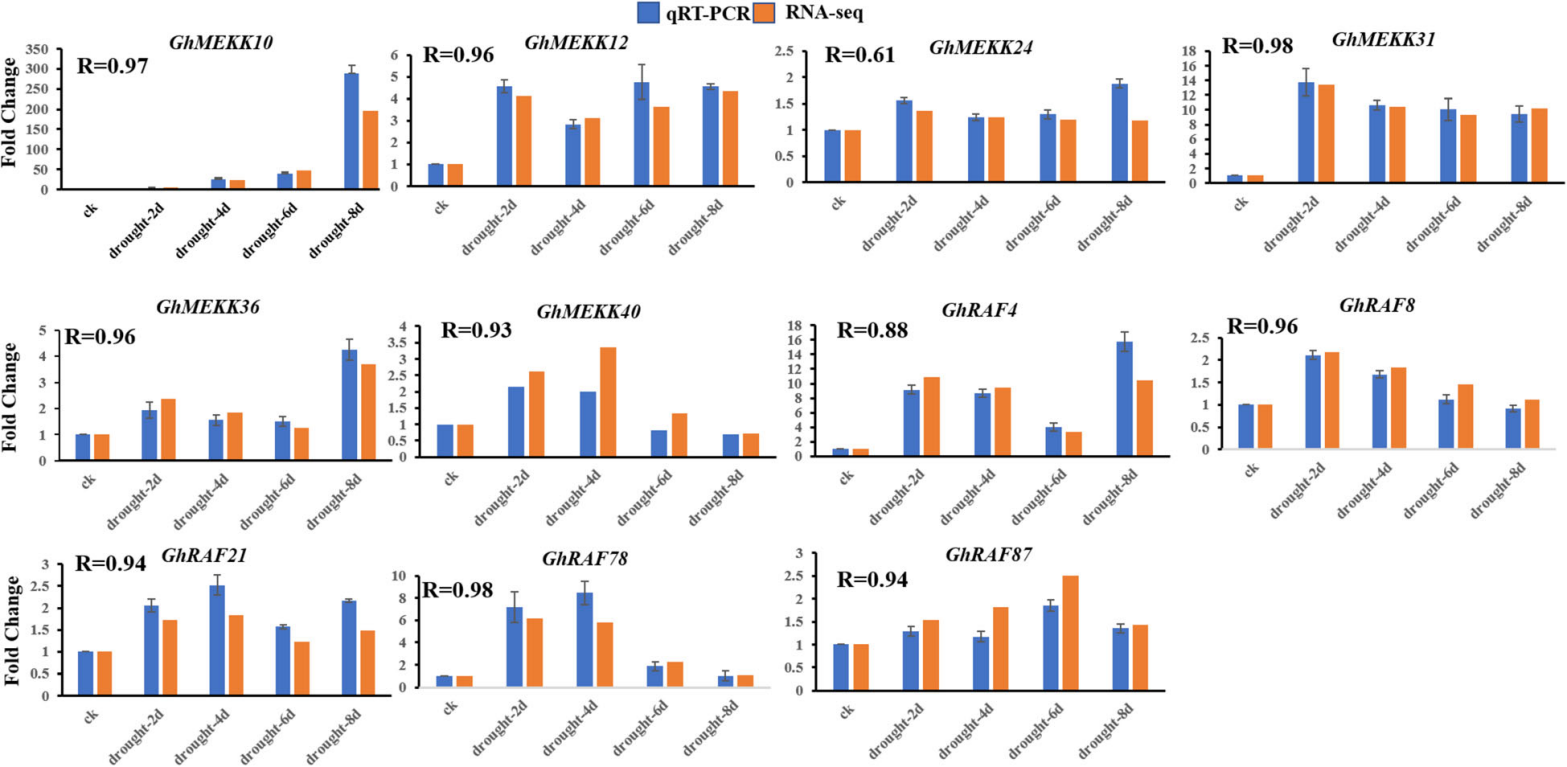
Quantitative RT-PCR and RNA-seq analysis of expressions of eleven *GhMAPKKKs* in cotton under drought treatments. The fold change calculated from quantitative RT-PCR analysis (blue bars) was compared with fold change from RNA-seq analysis (Orange bars). The R value represents the correlation coefficient. Error bars denote the standard deviation calculated from three independent experiments. *GhMEKK10* (Ghir_A08G021040), *GhMEKK12* (Ghir_A10G000750), *GhMEKK24* (Ghir_D02G008300), *GhMEKK31* (Ghir_D05G023540), *GhMEKK36* (Ghir_D10G001500), *GhMEKK40* (Ghir_D11G020530), *GhRAF4* (Ghir_A02G000730), *GhRAF8* (Ghir_A02G019080), *GhRAF21* (Ghir_A07G000080), *GhRAF78* (Ghir_D10G015940), *GhRAF87* (Ghir_D13G008130)

To confirm the results of transcriptome data, we further detected the transcript abundance of *GhMAPKKKs* in cotton seedlings under drought treatment by quantitative RT-PCR analysis. As shown in Fig. 7, the most genes examined were increased in cotton seedlings after drought treatment for 2–8 days, consistent with the transcriptome data. Under drought stress, many genes exhibited similar expression patterns. For instance, the transcripts of *GhMEKK40, GhRAF8, GhRAF21* and *GhRAF78* were largely accumulated in cotton seedlings under 2–4 days drought, but their expression levels were decreased in cotton after the subsequent 6–8 days drought. The expressions of *GhMEKK10*, *GhMEKK24*, *GhMEKK36* and *GhRAF4* were increased gradually in cotton under drought stress, reaching the peak value at 8th day. The above results suggest that *GhMAPKKKs* may be involved in cotton response and resistance to drought stress.

### Silencing *GhRAF4* and *GhMEKK*12 compromises cotton resistance to drought stress

**Fig. 8:**
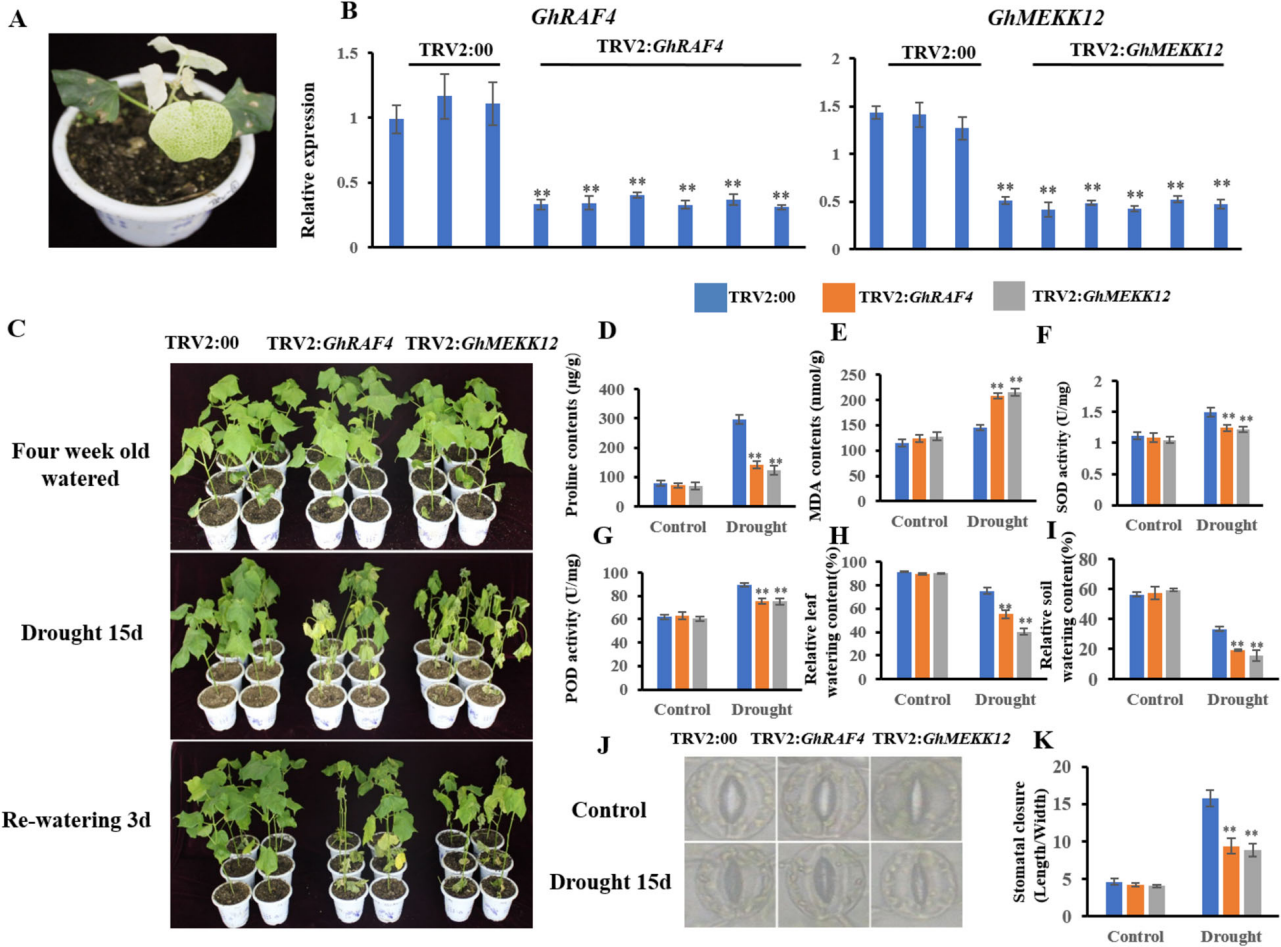
Silencing of *GhRAF4* and *GhMEKK12* compromises cotton tolerance to drought stress. **a** A positive control plant. **b** Quantitative RT-PCR analysis of expressions of *GhRAF4* and *GhMEKK12* in mock plants (TRV2:00) and VIGS plants (TRV2:GhRAF4 and TRV2:GhMEKK12). The cotton *UBI1* gene was used as internal control. Error bars denote the standard deviation calculated from three independent experiments. **c** Phenotype of mock plants and VIGS plants under drought stress. **d** – **i** Several physiological indexes measured in the target gene-silenced plants and mock plants under normal growing condition (control) and drought treatments. **d** Proline content. **e** MDA content. **f** SOD activity. **g** POD activity. **h** relative leaf watering content. **i** relative soil watering content. **j** Images of stoma in the target gene-silenced plants and mock plants under normal growing conditions (control) and drought treatment. **k** Comparative stomatal aperture measurements. Ratio of stomata length to width was determined in the target gene-silenced plants and mock plants under normal growing conditions (control) and drought treatment. Error bars denote the standard deviation calculated from three independent experiments. Asterisks represent Student’s t-test in statistical analysis for significant differences: *P < 0.05; **P < 0.01

Based on transcriptional levels of *GhMAPKKKs* in cotton under drought stress, we inferred that some *GhMAPKKKs* may take part in cotton response to drought stress. So, we employed VIGS technology to study the function of *GhMAPKKKs* in cotton under drought stress. As the transcription levels of *GhRAF4* and *GhMEKK12* are remarkably enhanced in cotton under drought treatment (Fig. 7), we selected the two genes for the VIGS experiments. After infection for 10 days, the plants with *TRV2:GhCLA* displayed the albino phenotype (Fig. 8a), indicating success of the VIGS experiment. Quantitative RT-PCR analysis revealed that the expression of *GhRAF4* and *GhMEKK*12 was significantly suppressed in the *TRV2:GhRAF4* and *TRV2:GhMEKK12* plants, compared with the controls (Fig. 8b). Four weeks later, the target gene-silenced (*TRV2:GhRAF4* and *TRV2:GhMEKK12*) and control (*TRV2:00*) plants were treated by drought stress. After 15 days of water-deficit treatment, the *TRV2:GhRAF4* and *TRV2:GhMEKK12* plants showed more severe wilting than the controls (Fig. 8c). Further analyses showed that the survival rates of the target gene silenced plants were significantly different from the control plants after re-watering. After re-watering for 3 days, the growth status of control plants gradually became normally, but only 66% TRV2:*GhRAF4* and 50% TRV2:*GhMEKK12* plants were survived (Fig. 8c). Additionally, we measured several physiological indexes including MDA and proline contents, and SOD and POD activities under drought treatment and normal growth conditions. As shown in Fig. 8d-g, under normal growth condition, *TRV2:GhRAF4* and *TRV2:GhMEKK12* plants didn’t show any difference compared with *TRV2:00* control plants. However, after water-withholding for 15 days, MDA content was increased, but the proline content and SOD and POD activities were reduced in TRV2:*GhRAF4* and *TRV2:GhMEKK12* plants, compared with those in *TRV2:00* control plants. Furthermore, the relative water contents in soil and leaves were no significant difference between the target gene silenced plants and the controls under normal growth condition. On the contrary, the relative water contents in both leaves and soil of target gene silenced plants were remarkably lower than those in control plants after drought treatment (Fig. 8h-i). When plants grew under normal condition, the stomatal aperture status of the target gene silenced plants is similar to that of the controls. Under drought treatment, however, the stoma of the target gene silenced plants opened larger than those of control plants (Fig. 8j-k). Collectively, the above results indicated that both osmotic adjustment ability and drought tolerance of the TRV2:*GhRAF4* and TRV2:*GhMEKK12* plants may be weakened owing to the *GhRAF4* or *GhMEKK12* gene silence.

## Discussion

*MAPKKK* gene family has been extensively analyzed in some plant species [15, 18, 22]. However, information about cotton *MAPKKK* gene family is relatively scarce so far. In this study, we used the published Arabidopsis MAPKKKs as queries to search upland cotton (*Gossypium hirsutum*) genome database, and identified 157 *GhMAPKKKs* in cotton genome. Phylogenetic analysis indicated the *GhMAPKKKs* can be divided into three groups (including 87 RAFs, 46 MEKKs and 24 ZIKs). Gene structure and motif composition analyses revealed that most GhMAPKKKs within the same group possess similar gene structure and motif distribution. However, some ZIKs and MEKKs also have particular motif composition, while some MEKK genes contain no intron in the open reading frame. The gene duplication events are significant in plant genomic variation, leading to the generation of new genes and genetic regulation pathways. The gene duplication (including tandem gene duplication and segmental gene duplication) is the major driver of gene family enlargement [23]. Our analysis showed that segmental duplication is a predominant driving force that contributed to the expansion of *GhMAPKKKs*.

In order to further understand the possible functions of *GhMAPKKK*s in cotton under different environmental stresses, we surveyed the cis-element distribution in promoter regions of *GhMAPKKK*s, and observed a huge number of cis-elements related to plant abiotic stress responses, suggesting that *GhMAPKKKs* may participate in regulation of cotton against abiotic stresses.

For the sake of better understanding of *GhMAPKKK*s in cotton growth and abiotic stress tolerance, we investigated the expression of *GhMAPKKK*s in various cotton tissues under normal condition and several abiotic stresses. The expression profiles of *GhMAPKKK* genes in leaves under drought stress were obtained from the transcriptome data in our lab, and we also detected the transcription levels of *GhMAPKKK*s under drought stresses by quantitative RT-PCR to confirm the previous transcriptome data. The transcriptome data and quantitative RT-PCR analysis revealed that the expression of most *GhMAPKKKs* is increased in cotton under water-deficit treatment, indicating these genes might be related to cotton drought response and resistance.

To investigate the roles of *GhMAPKKKs* in cotton drought tolerance, silencing of two *GhMAPKKKs*, *GhRAF4* and *GhMEKK*12, was carried out. The results showed that silencing *GhRAF4* and *GhMEKK*12 compromises cotton resistance to drought stress. Furthermore, we measured several physiological indexes (including MDA and proline contents, SOD and POD activities and leaf and soil relative water contents) under drought stress. The experimental results indicated that activities of SOD and POD were lower in the VIGS-silenced plants under drought stress, compared with the controls. The content of proline in the silenced plants was lower, whereas MDA content in the silenced plants was higher than those in the controls under drought stress. Similarly, the relative water content in leaves of the silenced plants as well as in soil was lower than that in the controls under drought stress. Drought stress can result in excessive production of reactive oxygen species (ROS), which brings about peroxidation of membrane lipid, disturbed physiological processes and programmed cell death [24, 25]. Therefore, removing superabundant ROS is a crucial process for plants defense to drought stress [1]. SOD and POD are essential enzymes in ROS-scavenging system, and their activities are increased in plants under drought stress [26]. Here, the higher MDA content, together with the lower SOD and POD activities in the VIGS-silenced cotton, suggest that the ability to remove ROS may be impaired. Similarly, previous studies also reported that *MAPKKKs* are related to plant drought resistance by modulating accumulation of ROS [27, 28]. Proline is known as an innoxious osmolyte and participates in osmotic regulation of the plant cell. To maintain the stability of protoplast colloid, the proline is accumulated to increase the osmotic pressure of plant cells under drought stress [29]. In this study, drought stress induced accumulation of proline in cotton, and thereby enhanced plant osmotic adjustment ability. The lower accumulation of proline in the VIGS-silenced cotton suggests the osmotic adjustment ability of these plants was weakened by silencing MAPKKK genes. Under drought stress, plants often reduce the opening degree of stoma to limit water loss in cells [30–32]. In this study, our data revealed that the stoma of the VIGS-silenced cotton plants opened wider than the controls under drought stress, suggesting that silencing *GhRAF4* and *GhMEKK12* influence the closure of stoma and thereby increase the water loss from plants.

## Conclusion

In this study, we comprehensively determined the evolutionary relationship, gene structure, motif distribution, cis-element dispersion and expression of *GhMAPKKKs* in cotton under normal condition and various abiotic stresses. The data indicated that the sequence features of *GhMAPKKKs* are both conservative and diverse. Besides, the roles of two *GhMAPKKK* genes (*GhRAF4* and *GhMEKK12*), whose expressions are significantly induced in cotton under drought stress, were investigated by VIGS technology. Silencing *GhRAF4* and *GhMEKK12* in cotton resulted in the increased plant sensitivity to drought stress. Thus, our data presented here may build up a good foundation for further investigating the roles of *GhMAPKKKs* in cotton response to drought stress in details.

## Methods

### Plant materials and growth conditions

Cotton (*Gossypium hirsutum* cv. Coker312) seeds provided by our lab were surface-sterilized with 10% hydrogen peroxide for 1.5 h, followed by washing three times with sterile water. The sterilized seeds were soaked in sterile water for 10–12 h, and then planted in pots and grew in a growth room under controlled conditions (25 °C, 16 h light/8 h dark). After approximately 10 days of growth, cotton seedlings with fully expanded cotyledons were used to conduct virus-induced gene silencing (VIGS) experiment.

### Sequence retrieval of *GhMAPKKKs*

The DNA and protein sequences of GhMAPKKKs were searched in cotton (*G. hirsutum* L., cultivar TM-1 (AD1)) genome database (https://www.cottongen.org). To identify *GhMAPKKKs*, the Arabidopsis MAPKKK proteins were employed as queries to search cotton (*G. hirsutum*) database. Then, to prove the accuracy of the blast results, all predicted protein sequences were subjected to InterproScan program and Pfam tools (http://www.sanger.ac.uk/software/pfam) to verify the presence of kinase domain [33]. Subsequently, we estimated the basic properties of cotton MAPKKK proteins using ProtParam tool (http://www.expasy.org/tools/protparam.html), and predicted the subcellular localization of cotton MAPKKKs using TargetP 1.1 (http://www.cbs.dtu.dk/services/TargetP/) and WoLF PSORT (http://wolfpsort.seq.cbrc.jp) programs [34, 35].

### Phylogenetic analysis

The MAPKKK protein sequences of Arabidopsis and cotton were used for multiple sequence alignments using Cluster X software. Subsequently, we constructed the unrooted phylogenetic tree based on the results of multiple sequence alignment using MEGA 6.0 software, and then the phylogenetic tree was submitted to ITOL (http://itol.embl.de/) to form the interactive tree.

### Gene structure analysis and conserved motif identification

The MAPKKK genomic sequences and their corresponding coding regions retrieved from cotton (*G. hirsutum*) genome were sent to Gene Structure Display Server (http://gsds.cbi.pku.edu.cn/) to investigate their exon/intron distribution [36]. All MAPKKK proteins were subjected to Motif Elicitation (MEME) online program (http://meme.sdsc.edu/meme/intro.html) to predict conserved motif. The InterProScan (http://www.ebi.ac.uk/Tools/InterPro-Scan/) was used to annotate the identified motifs [37].

### Chromosomal location and gene duplication analysis

The nucleotide sequences of these *GhMAPKKKs* were served as query for BLASTN to search cotton (*G. hirsutum*) genome database for gaining chromosomal location information. Next, we detected gene duplication events of *GhMAPKKKs* based on principle described in previous studies: (1) the alignment covers > 70% of the longer gene; (2) the aligned region has an identity > 70% [38–40]. The Circos-0.69 Software was used to visualize chromosomal location and gene duplication [41]. The Blast version 2.2.9 was used to identify orthologous gene pairs among the genomes of upland cotton and its ancestral A and D diploid cotton species [42]. Then, MCscanX was employed to identify homologous regions [43].

### Cis-element distribution in promoter regions of *GhMAPKKKs*

In order to determine cis-elements arrangement in promoter of *GhMAPKKKs*, we retrieved 2 kb upstream sequence of the transcriptional start site of *GhMAPKKKs* and submitted them to PLACE database (http://www.dna.affrc.go.jp/PLACE/) to investigate cis-element distribution.

### Expression analysis of *GhMAPKKKs* in cotton tissues under normal condition and different abiotic stresses

We downloaded the corresponding transcriptomic data of cotton (*G. hirsutum*) TM-1 from NCBI (https://www.ncbi.nlm.nih.gov/sra/?term=PRJNA248163). The reads were mapped to the cotton reference genome (v2.1) using TopHat2, and gene expression quantification was performed with Cufflinks (http://cole-trapnell-lab.github.iocufflinks/) [44].

### Analysis of expression of *GhMAPKKKs* under drought stress

In previous study, we carried out a transcriptome sequencing (RNA-seq) for drought-treated cotton [21]. Illumina reads were mapped to the reference TM-1 genome (v2.1) using TopHat 2.1.1 and quantification of gene expression was performed with Cufflinks version 2.2.1 (http://cole-trapnell-lab.github.iocufflinks/) by utilizing the GTF annotation file [44].

For detecting the expression of *GhMAPKKKs* in cotton under drought stress, quantitative RT-PCR analysis was employed. Cotton (cv. J13) seedlings grew in soil and were subjected to drought treatment. Then the leaves of these seedlings were collected at 0, 2, 4, 6, 8 days after drought stress for RNA extraction. The quantitative RT-PCR analysis was performed using gene-specific primers (Additional file 1 Table S4). Relative expression value of candidate genes was calculated with the 2 − ∆Ct method, and the housekeeping gene *GhUBI1* (EU604080) was used as an internal control. The experiments were performed with three biological replicates.

The fold change of gene expression from transcriptome data and quantitative RT-PCR analysis is the ratio of the gene expression value in cotton under drought treatment vs. the same gene expression value under normal condition (control) [45]. Then, correlation coefficient R value is calculated by using CORREL formula of Excel software.

### Vector construction and procedure for VIGS in cotton

The virus-induced gene silencing (VIGS) experiment was carried out by the method as previously described [46]. The fragments (400 bp) of *GhRAF4* and *GhMEKK12* were inserted into TRV2 vector to generate TRV2:*GhRAF4* and TRV2:*GhMEKK12* constructs, respectively. All the relevant primers were listed in Additional file 1 Table S3. The TRV1, TRV2:00, TRV2*:GhRAF4*, TRV2:*GhMEKK12* and TRV2*:CLA1* (cloroplastos alterados 1) constructs were transferred into *Agrobacterium tumefaciens* strain GV3101. Then, *A. tumefaciens* with TRV1 and *A. tumefaciens* containing TRV2:00, TRV2:*GhRAF4*, TRV2:*GhMEKK12* or TRV2:CLA1 were mixed in equal amounts and incubated at 28 °C for 3 h. The mixed agrobacterium solution was infiltrated into the cotyledons of 10-day-old cotton seedlings to generate the control (TRV:00) and *GhRAF4* (TRV:*GhRAF4*) and *GhMEKK12* (TRV:*GhMEKK12*) silenced cotton plants. The TRV:*CLA1* was used as a positive control. When the TRV:*CLA1* plants appear albino phenotype, indicating that the gene has been silenced. Subsequently, the expression of *GhRAF4* and *GhMEKK12* in the TRV:*GhRAF4* and TRV:*GhMEKK12* silenced plants and wild type controls were detected by quantitative RT-PCR analysis. Then the four-week-old silenced cotton plants and wild type controls were subjected to water-deficit treatment. The experiments were performed with three independent biological replicates.

### Expression analysis of *GhRAF4* and *GhMEKK*12 in VIGS and control plants

To investigate gene expression in TRV2:*GhRAF4* and TRV2:*GhMEKK*12 seedlings, the third true leaves of these cotton seedlings were used to extract total RNA. Then, the RNA was reversely transcribed into cDNA using an RNA reverse transcription kit (AMV, version 3.0, TaKaRa, Shuzo, Otsu, Japan). The quantitative PCR was performed using gene-specific primers (Additional file 1 Table S4). The housekeeping gene *GhUBI1* (EU604080) was used as an internal control. The experiments were performed with three biological replicates.

### Determination of drought stress-related physiological parameters

Malondialdehyde (MDA) content was determined from 0.1 g cotton leaf tissues by using MDA Quantification Assay Kit (Nanjing Jiancheng Bioengineering Institute, Nanjing, China). For quantification of proline content, 0.1 g samples of cotton leaves were prepared and followed the procedure as described by the manufacturer of Proline Quantification Assay Kit (Nanjing Jiancheng Bioengineering Institute, Nanjing, China). The relative watering content of leaves was measured as the method described by Barrs and Weatherley [47]. The measurement of peroxidase (POD) and superoxide dismutase (SOD) enzyme activities in the stressed plants and controls was performed with described by the manufacturer of Peroxidase (POD) assay kit and Superoxide Dismutase (SOD) assay kit (Nanjing Jiancheng Bioengineering Institute, Nanjing, China). Stomatal aperture was observed in leaves of cotton under normal condition and drought treatment by microscopy, and the ratio of stomatal length to width was measured (*n* > 50 stomata per sample).

## Supplementary information

**Additional file 1 Table S1.** Characterization of upland cotton (*Gossypium hirsutum*) MAPKKK family. **Table S2.** Orthologous MAPKKK gene pairs of *Gossypium hirsutum*, *G. arboreum*, and *G. raimondii.***Table S3.** Primers used in vector construction and quantitative RT-PCR analysis. **Figure S1.** Phylogenetic relationship of GhMAPKKKs and AtMAPKKKs. **Figure S2.** Analysis of *GhMAPKKK* promoters.

## Data Availability

All data used during the current study are included in this published article or are available from the corresponding author on reasonable request.

## Abbreviations

MAPKKK: Mitogen-activated protein kinase kinase kinase
PEG: Polyethylene glycol
VIGS: Virus-induced gene silencing
ABRE: Abscisic acid -responsive element
DRE: Dehydration responsive element
RT-PCR: Reverse transcription-polymerase chain reaction
MDA: Malondialdehyde
SOD: Superoxide dismutase
POD: Peroxidase
ROS: Reactive oxygen species

## References

[1] Paterson AH, Wendel JF, Gundlach H, Guo H, Jenkins J, Jin D, Yoo MJ (2012). Repeated polyploidization of Gossypium genomes and the evolution of spinnable cotton fibres. Nature.

[2] Soltis DE, Soltis PS, Tate JA (2004). Advances in the study of polyploidy since plant speciation. New Phytol.

[3] Massacci A, Nabiev SM, Pietrosanti L, Nematov SK, Chernikova TN, Thor K, Leipner J (2008). Response of the photosynthetic apparatus of cotton (*Gossypium hirsutum*) to the onset of drought stress under field conditions studied by gas-exchange analysis and chlorophyll fluorescence imaging. Plant Physiol Biochem.

[4] Asai T, Tena G, Plotnikova J, Willmann MR, Chiu WL, Gomez-Gomez L, Sheen J (2002). MAP kinase signalling cascade in Arabidopsis innate immunity. Nature.

[5] Lewis TS, Shapiro PS, Ahn NG (1998). Signal transduction through MAP kinase cascades. Adv Cancer Res.

[6] Nishihama R, Banno H, Shibata W, Hirano K, Nakashima M, Usami S, Machida Y (1995). Plant homologues of components of MAPK (mitogen-activated protein kinase) signal pathways in yeast and animal cells. Plant Cell Physiol.

[7] Pitzschke A, Schikora A, Hirt H (2009). MAPK cascade signaling networks in plant defence. Curr Opin Plant Biol.

[8] Liu Y, Schiff M, Dinesh-Kumar SP (2004). Involvement of MEK1 MAPKK, NTF6 MAPK, WRKY/MYB transcription factors, COI1 and CTR1 in N-mediated resistance to tobacco mosaic virus. Plant J.

[9] Li F, Li M, Wang P, Cox KL, Duan L, Dever JK, He P (2017). Regulation of cotton (Gossypium hirsutum) drought responses by mitogen-activated protein (MAP) kinase cascade-mediated phosphorylation of *GhWRKY59*. New Phytol.

[10] Colcombet J, Hirt H (2008). Arabidopsis MAPKs: a complex signaling network involved in multiple biological processes. Biochem J.

[11] Ning J, Li X, Hicks LM, Xiong L (2010). A Raf-like MAPKKK gene DSM1 mediates drought resistance through reactive oxygen species scavenging in rice. Plant Physiol.

[12] Shou H, Bordallo P, Fan JB, Yeakley JM, Bibikova M, Sheen J, Wang K (2004). Expression of an active tobacco mitogen-activated protein kinase kinase kinase enhances freezing tolerance in transgenic maize. Proc Natl Acad Sci.

[13] Li Y, Cai H, Liu P, Wang C, Gao H, Wu C, Zheng C (2017). Arabidopsis MAPKKK18 positively regulates drought stress resistance via downstream MAPKK3. Biochem Biophys Res Commun.

[14] Ichimura K, Shinozaki K, Tena G, Sheen J, Henry Y, Champion A, Heberle-Bors E (2002). Mitogen-activated protein kinase cascades in plants: a new nomenclature. Trends Plant Sci.

[15] Rao KP, Richa TAMBI, Kumar KUNDAN, Raghuram BADMI, Sinha AK (2010). In silico analysis reveals 75 members of mitogen-activated protein kinase kinase kinase gene family in rice. DNA Res.

[16] Wang M, Yue H, Feng K, Deng P, Song W, Nie X (2016). Genome-wide identification, phylogeny and expressional profiles of mitogen activated protein kinase kinase kinase (MAPKKK) gene family in bread wheat (*Triticum aestivum* L.). BMC Genom.

[17] Kong X, Lv W, Zhang D, Jiang S, Zhang S, Li D (2013). Genome-wide identification and analysis of expression profiles of maize mitogen-activated protein kinase kinase kinase. PLoS One.

[18] Wang J, Pan C, Wang Y, Ye L, Wu J, Chen L, Lu G (2015). Genome-wide identification of MAPK, MAPKK, and MAPKKK gene families and transcriptional profiling analysis during development and stress response in cucumber. BMC Genomics.

[19] Wang M, Tu L, Yuan D, Zhu D, Shen C, Li J, Ye Z (2019). Reference genome sequences of two cultivated allotetraploid cottons, Gossypium hirsutum and Gossypium barbadense. Nat Genet.

[20] Li F, Fan G, Lu C, Xiao G, Zou C, Kohel RJ, Liang X (2015). Genome sequence of cultivated upland cotton (Gossypium hirsutum TM-1) provides insights into genome evolution. Nat Biotechnol.

[21] Chen Y, Liu ZH, Feng L, Zheng Y, Li DD, Li XB (2013). Genome-wide functional analysis of cotton (Gossypium hirsutum) in response to drought. PLoS One.

[22] Xu J, Zhang S (2015). Mitogen-activated protein kinase cascades in signaling plant growth and development. Trends Plant Sci.

[23] Flagel LE, Wendel JF (2009). Gene duplication and evolutionary novelty in plants. New Phytol.

[24] Fu J, Huang BJE, Botany E (2001). Involvement of antioxidants and lipid peroxidation in the adaptation of two cool-season grasses to localized drought stress. Environ Exp Bot.

[25] Sugimoto M, Oono Y, Gusev O, Matsumoto T, Yazawa T, Levinskikh MA, Sychev VN, Bingham GE, Wheeler R, Hummerick M (2014). Genome-wide expression analysis of reactive oxygen species gene network in Mizuna plants grown in long-term spaceflight. BMC Plant Biol.

[26] Luis A, Corpas FJ, López-Huertas E, Palma JM (2018). Plant superoxide dismutases: function under abiotic stress conditions. Antioxidants and antioxidant enzymes in higher plants.

[27] Pitzschke A, Djamei A, Bitton F, Vhirt H (2009). A major role of the MEKK1–MKK1/2–MPK4 pathway in ROS signalling. Mol Plant.

[28] Jalmi SK, Sinha A (2015). K ROS mediated MAPK signaling in abiotic and biotic stress-striking similarities and differences. Front Plant Sci.

[29] Nahar K, Ullah SM. Drought stress effects on plant water relations, growth, fruit quality and osmotic adjustment of tomato (Solanum lycopersicum) under subtropical condition. Asian J Agric Horticultural Res. 2018;1(2):1–14.

[30] Levin AD, Williams LE, Matthews MA (2020). A continuum of stomatal responses to water deficits among 17 wine grape cultivars (Vitis vinifera). Funct Plant Biol.

[31] Huang L, Chen L, Wang L, Yang Y, Rao Y, Ren D, Zhang G. A Nck-associated protein 1-like protein affects drought sensitivity by its involvement in leaf epidermal development and stomatal closure in rice. Plant J. 2019;98(5):884–97. 10.1111/tpj.14288 PMC6849750 30771248

[32] Bang SW, Lee DK, Jung H, Chung PJ, Kim YS, Choi YD, Kim JK (2019). Overexpression of OsTF1L, a rice HD-zip transcription factor, promotes lignin biosynthesis and stomatal closure that improves drought tolerance. Plant Biotechnol J.

[33] Gasteiger E, Hoogland C, Gattiker A, Wilkins MR, Appel RD, Bairoch A (2005). Protein identification and analysis tools on the ExPASy server. The proteomics protocols handbook.

[34] Horton P, Park KJ, Obayashi T, Fujita N, Harada H, Adams-Collier CJ, Nakai K (2007). WoLF PSORT: protein localization predictor. Nucleic Acids Res.

[35] Emanuelsson O, Nielsen H, Brunak S, Von Heijne G (2000). Predicting subcellular localization of proteins based on their N-terminal amino acid sequence. J Mol Biol.

[36] Guo AY, Zhu QH, Chen X, Luo JC (2007). GSDS: a gene structure display server. Yi chuan.

[37] De Castro E, Sigrist CJ, Gattiker A, Bulliard V, Langendijk-Genevaux PS, Gasteiger E, Hulo N (2006). ScanProsite: detection of PROSITE signature matches and ProRule-associated functional and structural residues in proteins. Nucleic Acids Res.

[38] Yang S, Zhang X, Yue JX, Tian D, Chen JQ (2008). Recent duplications dominate NBS-encoding gene expansion in two woody species. Mol Gen Genomics.

[39] Gu Z, Cavalcanti A, Chen FC, Bouman P, Li WH (2002). Extent of gene duplication in the genomes of Drosophila, nematode, and yeast. Mol Biol Evol.

[40] Ma J, Wang Q, Sun R, Xie F, Jones DC, Zhang B (2014). Genome-wide identification and expression analysis of TCP transcription factors in Gossypium raimondii. Sci Rep.

[41] Krzywinski M, Schein J, Birol I, Connors J, Gascoyne R, Horsman D, Jones SJ, Marra MA (2009). Circos: an information aesthetic for comparative genomics. Genome Res.

[42] Altschul SF, Gish W, Miller W, Myers EW, Lipman DJ (1990). Basic local alignment search tool. J Mol Biol.

[43] Wang Y, Tang H, DeBarry JD, Tan X, Li J, Wang X, Kissinger JC (2012). MCScanX: a toolkit for detection and evolutionary analysis of gene synteny and collinearity. Nucleic Acids Res.

[44] Ghosh S, Chan CKK (2016). Analysis of RNA-Seq data using TopHat and Cufflinks. Plant Bioinformatics.

[45] Hussain A, Mun BG, Imran QM, Lee SU, Adamu TA, Shahid M, Kim KM, Yun BW (2016). Nitric Oxide Mediated Transcriptome Profiling Reveals Activation of Multiple Regulatory Pathways in *Arabidopsis thaliana*. Front Plant Sci.

[46] Gao W, Long L, Xu L, Lindsey K, Zhang X, Zhu L (2016). Suppression of the homeobox gene HDTF1 enhances resistance to Verticillium dahliae and Botrytis cinerea in cotton. J Integr Plant Biol.

[47] Barrs HD, Weatherley PE (1962). A re-examination of the relative turgidity technique for estimating water deficits in leaves. Aust J Biol Sci.

