# Supplementary material for: Genome-wide identification and functional characterization of cotton (Gossypium hirsutum) MAPKKK gene family in response to drought stress
Source: BMC Plant Biol. 2020 May 14;20:217. doi: 10.1186/s12870-020-02431-2 (PMC7227315; doi:10.1186/s12870-020-02431-2)
Supplement: Supplementary file 1 — Additional file 1 Table S1. Characterization of upland cotton (Gossypium hirsutum) MAPKKK family. Table S2. Orthologous MAPKKK gene pairs of Gossypium hirsutum, G. arboreum, and G. raimondii.Table S3. Primers used in vector construction and quantitative RT-PCR analysis. Figure S1. Phylogenetic relationship of GhMAPKKKs and AtMAPKKKs. Figure S2. Analysis of GhMAPKKK promoters. [file 12870_2020_2431_MOESM1_ESM.pdf]

Additional file 1 (Tables and Figures)

Table S1. Characterization of upland cotton (*Gossypium hirsutum*) MAPKKK family.

Gene name	Gene symbol	Protein Lengh(aa)	MW(kDa)	pI	Chr	Chr location	subcellular location
GhRAF1	Ghir_A01G013020	790	86.94	5.71	A01	59116528-59124508	nucleus
GhRAF2	Ghir_A01G016390	640	73.17	7.46	A01	106268030-106276569	mitochondria
GhRAF3	Ghir_A02G000450	339	37.98	7.48	A02	265264-268155	cytoplasm
GhRAF4	Ghir_A02G000730	472	53.59	6.65	A02	533011-536829	nucleus
GhRAF5	Ghir_A02G003480	383	42.49	9.52	A02	3913633-3915990	cytoplasm
GhRAF6	Ghir_A02G005560	737	81.69	7.5	A02	7937650-7943308	nucleus
GhRAF7	Ghir_A02G005570	740	82.04	7.1	A02	7967940-7973147	nucleus
GhRAF8	Ghir_A02G019080	531	59.81	9.08	A02	107728162-107734200	cytoplasm
GhRAF9	Ghir_A03G011560	429	48.44	4.72	A03	66726431-66731117	nucleus
GhRAF10	Ghir_A04G007330	851	94.44	6.72	A04	55875639-55885335	chloroplast
GhRAF11	Ghir_A04G013720	560	63.32	6.7	A04	82511312-82523191	cytoplasm
GhRAF12	Ghir_A05G000660	1101	122.21	6.02	A05	791306-798656	nucleus
GhRAF13	Ghir_A05G004450	931	103.68	5.64	A05	4095618-4103212	nucleus
GhRAF14	Ghir_A05G006850	419	46.76	5.87	A05	6210586-6215373	cytoplasm
GhRAF15	Ghir_A05G018630	504	57.03	5.11	A05	17620458-17627531	cytoplasm
GhRAF16	Ghir_A05G036930	352	39.79	5.67	A05	97148360-97152224	cytoplasm
GhRAF17	Ghir_A05G037260	374	41.97	6.36	A05	98076719-98080529	nucleus
GhRAF18	Ghir_A06G003070	915	102.20	5.06	A06	3871954-3879898	nucleus
GhRAF19	Ghir_A06G005200	1361	147.82	6.54	A06	8680343-8685948	nucleus
GhRAF20	Ghir_A06G016490	371	42.45	5.05	A06	114049596-114052061	mitochondria
GhRAF21	Ghir_A07G000080	415	46.36	9.3	A07	122914-127571	cytoplasm
GhRAF22	Ghir_A07G002310	844	94.54	5.41	A07	2497285-2504622	cytoplasm
GhRAF23	Ghir_A07G023620	379	42.39	5	A07	95826552-95830147	nucleus
GhRAF24	Ghir_A07G024120	354	40.03	7.82	A07	96546518-96550072	cytoplasm
GhRAF25	Ghir_A08G007020	1110	122.58	6.85	A08	11163822-11175803	mitochondria
GhRAF26	Ghir_A08G009370	1136	125.95	9.42	A08	36606576-36615782	nucleus
GhRAF27	Ghir_A09G003240	351	39.65	5.78	A09	10448608-10451805	cytoplasm
GhRAF28	Ghir_A09G005960	1035	113.38	5.58	A09	45765254-45780842	nucleus
GhRAF29	Ghir_A09G007360	765	84.97	9.43	A09	54426475-54431583	nucleus
GhRAF30	Ghir_A09G014590	846	94.01	7.02	A09	69970458-69980083	chloroplast
GhRAF31	Ghir_A09G022480	470	53.03	6.3	A09	78659520-78665922	mitochondria
GhRAF32	Ghir_A09G022610	355	39.85	8.8	A09	78818941-78822312	cytoplasm
GhRAF33	Ghir_A09G023270	391	43.58	7.66	A09	79321195-79325067	cytoplasm
GhRAF34	Ghir_A09G024870	777	86.08	7.31	A09	80703148-80707529	nucleus
GhRAF35	Ghir_A10G000530	374	42.65	6.06	A10	359133-362546	mitochondria
GhRAF36	Ghir_A10G014920	472	53.49	9.29	A10	79999093-80009164	chloroplast

GhRAF37	Ghir_A10G017650	815	90.15	5.22	A10	98138758-98144602	plastid
GhRAF38	Ghir_A10G017660	775	85.74	7.85	A10	98138949-98144056	nucleus
GhRAF39	Ghir_A11G007400	383	42.65	9.55	A11	6471350-6476392	cytoplasm
GhRAF40	Ghir_A11G018110	576	65.33	7.83	A11	23594659-23601101	cytoplasm
GhRAF41	Ghir_A12G000340	390	43.49	8.55	A12	498790-502564	cytoplasm
GhRAF42	Ghir_A12G017420	437	49.15	5.65	A12	92993297-92998342	cytoplasm
GhRAF43	Ghir_A12G028160	1005	109.59	5.11	A12	106836116-106845511	chloroplast
GhRAF44	Ghir_A12G028660	405	44.83	8.75	A12	107354905-107359160	nucleus
GhRAF45	Ghir_A13G007800	422	47.24	6.43	A13	22199989-22209556	nucleus
GhRAF46	Ghir_D01G000200	552	62.23	6.47	D01	119887-126036	cytoplasm
GhRAF47	Ghir_D01G014020	859	94.98	6.58	D01	33463668-33473973	nucleus
GhRAF48	Ghir_D01G017900	640	73.22	9.08	D01	54254478-54262863	mitochondria
GhRAF49	Ghir_D02G000450	353	39.42	7.86	D02	292116-295007	cytoplasm
GhRAF50	Ghir_D02G000700	479	54.34	8.52	D02	557041-561510	nucleus
GhRAF51	Ghir_D02G003900	421	47.12	7.26	D02	4871769-4874033	vacuole
GhRAF52	Ghir_D02G005920	612	67.27	9.13	D02	7896079-7899981	nucleus
GhRAF53	Ghir_D02G013070	430	48.50	6.05	D02	43746961-43751699	nucleus
GhRAF54	Ghir_D03G000490	571	64.31	7.63	D03	287684-293745	cytoplasm
GhRAF55	Ghir_D04G005790	374	41.93	6.36	D04	9363047-9371442	nucleus
GhRAF56	Ghir_D04G006140	352	39.80	6.27	D04	9990673-9994944	cytoplasm
GhRAF57	Ghir_D04G011410	769	85.44	6.03	D04	36701936-36715894	chloroplast
GhRAF58	Ghir_D04G018140	581	65.69	5.68	D04	53642245-53651176	cytoplasm
GhRAF59	Ghir_D05G004560	931	103.38	7.9	D05	3654314-3661796	nucleus
GhRAF60	Ghir_D05G018650	546	61.82	7.59	D05	16164014-16171000	cytoplasm
GhRAF61	Ghir_D06G003070	915	102.18	9.17	D06	3677514-3685966	nucleus
GhRAF62	Ghir_D06G017360	371	42.43	8.11	D06	57749167-57752516	mitochondria
GhRAF63	Ghir_D07G000100	415	46.33	4.96	D07	179515-186775	chloroplast
GhRAF64	Ghir_D07G002320	937	104.13	8.16	D07	2398608-2406880	cytoplasm
GhRAF65	Ghir_D07G023720	407	45.62	9.43	D07	57351655-57355299	nucleus
GhRAF66	Ghir_D07G024190	354	39.98	7.02	D07	57953528-57957106	cytoplasm
GhRAF67	Ghir_D08G007100	1110	122.75	6.08	D08	9538439-9550399	mitochondria
GhRAF68	Ghir_D08G009250	1137	126.20	8.2	D08	19075396-19083848	nucleus
GhRAF69	Ghir_D09G003170	368	41.74	9.61	D09	9674313-9676427	cytoplasm
GhRAF70	Ghir_D09G005530	1038	113.70	7.76	D09	25995770-26013030	chloroplast
GhRAF71	Ghir_D09G007100	766	85.43	9.5	D09	31322296-31328509	nucleus
GhRAF72	Ghir_D09G021710	511	58.31	8.45	D09	49737805-49743942	plastid
GhRAF73	Ghir_D09G021860	355	39.85	8.05	D09	49864023-49867444	cytoplasm
GhRAF74	Ghir_D09G022500	391	43.62	5.05	D09	50337160-50340960	cytoplasm
GhRAF75	Ghir_D09G024040	782	86.48	5.3	D09	51636834-51641319	nucleus
GhRAF76	Ghir_D10G001280	374	42.58	9.49	D10	1044184-1047688	mitochondria
GhRAF77	Ghir_D10G012570	472	53.39	6.59	D10	20394725-20405455	chloroplast
GhRAF78	Ghir_D10G015940	407	45.59	6.12	D10	34491476-34496357	nucleus
GhRAF79	Ghir_D10G027360	822	91.39	6.52	D10	42627-48353	nucleus
GhRAF80	Ghir_D10G027370	822	91.39	7.85	D10	42627-48353	nucleus

GhRAF81	Ghir_D11G007370	377	42.05	8.15	D11	5956229-5961305	cytoplasm
GhRAF82	Ghir_D11G013250	391	43.65	9.12	D11	12324700-12328019	nucleus
GhRAF83	Ghir_D11G018170	575	65.08	6.55	D11	19660407-19666208	cytoplasm
GhRAF84	Ghir_D12G000320	390	43.51	4.76	D12	496884-500310	cytoplasm
GhRAF85	Ghir_D12G017630	407	45.66	6.51	D12	50376204-50380659	nucleus
GhRAF86	Ghir_D12G028810	395	44.04	6.92	D12	62344984-62348539	cytoplasm
GhRAF87	Ghir_D13G008130	422	47.36	5.13	D13	14566968-14572225	nucleus
GhMEKK1	Ghir_A01G007310	437	48.73	4.4	A01	12214003-12215349	chloroplast
GhMEKK2	Ghir_A01G001550	1392	153.45	6.38	A01	1347205-1364027	nucleus
GhMEKK3	Ghir_A02G007890	576	63.86	7.86	A02	14863551-14868958	nucleus
GhMEKK4	Ghir_A02G017190	338	37.24	4.8	A02	105635187-105636628	nucleus
GhMEKK5	Ghir_A03G003570	483	54.23	7.04	A03	5378191-5379713	chloroplast
GhMEKK6	Ghir_A05G017620	451	50.22	8.12	A05	16817349-16819236	chloroplast
GhMEKK7	Ghir_A05G023670	590	65.81	7.33	A05	23521490-23527510	mitochondria
GhMEKK8	Ghir_A05G039730	661	71.49	7.83	A05	104396747-104402568	nucleus
GhMEKK9	Ghir_A08G019150	898	96.61	5.83	A08	113804134-113811609	nucleus
GhMEKK10	Ghir_A08G021040	510	57.38	4.66	A08	116673745-116675288	chloroplast
GhMEKK11	Ghir_A09G006310	696	77.07	8.21	A09	48820595-48825206	nucleus
GhMEKK12	Ghir_A10G000750	446	49.88	5.16	A10	542297-544223	chloroplast
GhMEKK13	Ghir_A10G008980	336	37.06	8.89	A10	17413794-17415339	cytoplasm
GhMEKK14	Ghir_A10G009080	609	67.15	5.67	A10	17850579-17854710	chloroplast
GhMEKK15	Ghir_A11G005180	711	78.83	8.93	A11	4446012-4450377	nucleus
GhMEKK16	Ghir_A11G035820	359	40.06	7.85	A11	74237-75959	nucleus
GhMEKK17	Ghir_A11G028650	649	71.28	6.18	A11	110953343-110957688	chloroplast
GhMEKK18	Ghir_A12G000100	659	71.27	9.09	A12	128695-134251	nucleus
GhMEKK19	Ghir_A12G007320	724	79.50	8.34	A12	20022401-20027163	chloroplast
GhMEKK20	Ghir_A12G014480	1419	156.93	6.58	A12	85092129-85106151	nucleus
GhMEKK21	Ghir_A13G024620	480	53.88	4.87	A13	108139019-108140767	nucleus
GhMEKK22	Ghir_D01G001550	1390	153.24	6.42	D01	1117035-1129976	nucleus
GhMEKK23	Ghir_D01G007620	437	48.70	4.4	D01	10460363-10461748	chloroplast
GhMEKK24	Ghir_D02G008300	589	65.29	6.21	D02	13393209-13401017	nucleus
GhMEKK25	Ghir_D02G019480	1433	157.75	6.19	D02	64489810-64506546	nucleus
GhMEKK26	Ghir_D03G002430	338	37.27	4.74	D03	2289862-2291287	nucleus
GhMEKK27	Ghir_D03G015380	466	52.13	7.57	D03	47788539-47791494	chloroplast
GhMEKK28	Ghir_D04G003320	492	54.23	9.47	D04	4494122-4499940	mitochondria
GhMEKK29	Ghir_D05G010730	519	58.18	5.12	D05	9039986-9043494	nucleus
GhMEKK31	Ghir_D05G017600	328	36.08	5.65	D05	15286900-15287915	chloroplast
GhMEKK32	Ghir_D05G023540	590	65.77	5.8	D05	21265802-21271793	mitochondria
GhMEKK33	Ghir_D08G020070	886	95.34	4.71	D08	60648887-60653844	nucleus
GhMEKK34	Ghir_D08G021840	485	54.48	9.29	D08	63216185-63217671	chloroplast
GhMEKK35	Ghir_D09G004580	896	96.72	7.83	D09	17634778-17642198	nucleus
GhMEKK36	Ghir_D09G005940	666	73.65	5.52	D09	27714016-27718642	nucleus
GhMEKK37	Ghir_D10G001500	445	49.28	8.89	D10	1232613-1234017	chloroplast
GhMEKK38	Ghir_D10G010170	613	67.53	6.02	D10	12704209-12708378	chloroplast

GhMEKK39	Ghir_D10G010300	336	37.02	8.25	D10	12944719-12946243	cytoplasm
GhMEKK39	Ghir_D11G005120	711	78.85	8.82	D11	4146812-4151046	nucleus
GhMEKK40	Ghir_D11G020530	359	39.96	6.6	D11	24796013-24797663	nucleus
GhMEKK41	Ghir_D11G028840	663	72.81	6.63	D11	61257989-61262800	chloroplast
GhMEKK42	Ghir_D12G000070	657	71.09	6	D12	118968-123487	nucleus
GhMEKK43	Ghir_D12G007230	724	79.39	8.15	D12	13666895-13671597	chloroplast
GhMEKK44	Ghir_D12G009430	350	39.04	8.37	D12	30737030-30738538	nucleus
GhMEKK45	Ghir_D12G014650	1419	156.77	5.04	D12	45189485-45203561	nucleus
GhMEKK46	Ghir_D13G025390	400	44.60	5.14	D13	63120547-63122405	cytoplasm
GhZIK1	Ghir_A01G007760	294	33.51	4.76	A01	12963498-12965306	nucleus
GhZIK2	Ghir_A02G003230	295	83.12	5.06	A02	3711890-3715525	nucleus
GhZIK3	Ghir_A02G010480	593	68.47	5.54	A02	44849057-44852765	chloroplast
GhZIK4	Ghir_A05G039820	734	68.02	5.63	A05	104503240-104507236	nucleus
GhZIK5	Ghir_A07G006240	643	68.75	8.56	A07	7493603-7497360	nucleus
GhZIK6	Ghir_A08G013280	584	75.45	8.81	A08	95860535-95864059	nucleus
GhZIK7	Ghir_A09G025930	607	72.14	4.79	A09	81675593-81680150	nucleus
GhZIK8	Ghir_A10G015190	300	67.70	4.64	A10	83054890-83059051	nucleus
GhZIK9	Ghir_A11G023700	593	72.43	9.68	A11	71611997-71615920	nucleus
GhZIK10	Ghir_A11G027620	606	65.84	8.11	A11	108571185-108575022	nucleus
GhZIK11	Ghir_A12G025200	610	68.38	6.97	A12	104071155-104075057	nucleus
GhZIK12	Ghir_A13G024870	611	67.42	7.87	A13	108274667-108278662	nucleus
GhZIK13	Ghir_D01G008110	667	33.70	8.33	D01	11151704-11153578	nucleus
GhZIK14	Ghir_D02G003610	639	82.81	6.13	D02	4677447-4681187	nucleus
GhZIK15	Ghir_D04G003230	593	67.82	7.85	D04	4404068-4408048	nucleus
GhZIK16	Ghir_D07G006240	643	68.65	5.83	D07	6781781-6785762	nucleus
GhZIK17	Ghir_D08G014030	573	75.63	6.66	D08	48402157-48405717	nucleus
GhZIK18	Ghir_D09G025080	607	69.97	5.13	D09	52454940-52459320	nucleus
GhZIK19	Ghir_D10G012370	593	67.73	4.85	D10	19337073-19341117	nucleus
GhZIK20	Ghir_D11G024040	734	72.10	5.65	D11	43762327-43766202	nucleus
GhZIK21	Ghir_D11G027780	609	67.05a	5.79	D11	59353199-59356901	nucleus
GhZIK22	Ghir_D12G025200	609	68.57	7.66	D12	59149045-59153596	nucleus
GhZIK23	Ghir_D13G014800	668	34.31	5.99	D13	48319020-48320442	nucleus
GhZIK24	Ghir_D13G025640	621	67.44	4.8	D13	63270786-63274742	nucleus

Note: The DNA and protein sequences of GhMAPKKKs can be found in cotton (*G. hirsutum*) genome database (https://www.cottongen.org).

Table S2. Orthologous MAPKKK gene pairs of *Gossypium hirsutum*, *G. arboreum*, and *G. raimondii.*

Genes of At subgenome	Orthologous genes in G. arboreum	Genes of Dt subgenome	Orthologous genes of G. raimondii
GhMEKK1	Ga01G0882.1	*GhMEKK22*	Gorai.002G016000.1
GhMEKK2	Ga01G0174.1	*GhMEKK23*	Gorai.002G091000.1
GhMEKK3	Ga03G0881.1	*GhMEKK24*	Gorai.005G091700.1
GhMEKK4	Ga02G0227.1	*GhMEKK25*	Gorai.005G210100.1
GhMEKK5	Ga01G2525.1	*GhMEKK26*	Gorai.003G025400.1
GhMEKK6	Ga05G1835.1	*GhMEKK27*	Gorai.003G146500.1
GhMEKK7	Ga05G2467.1	*GhMEKK28*	Gorai.012G034500.1
GhMEKK8	Ga04G1803.1	*GhMEKK29*	Gorai.009G111600.1
GhMEKK9	Ga08G2155.1	*GhMEKK31*	Gorai.009G242600.1
GhMEKK10	Ga08G2357.1	*GhMEKK32*	Gorai.004G213200.1
GhMEKK11	Ga09G0760.1	*GhMEKK33*	Gorai.004G232400.1
GhMEKK12	Ga10G2971.1	*GhMEKK34*	Gorai.006G049500.1
GhMEKK13	Ga10G2072.1	*GhMEKK35*	Gorai.006G052900.1
GhMEKK14	Ga10G2060.1	*GhMEKK36*	Gorai.011G012900.1
GhMEKK15	Ga11G3597.1	*GhMEKK37*	Gorai.011G104700.1
GhMEKK16	Ga11G1935.1	*GhMEKK38*	Gorai.011G106000.1
GhMEKK17	Ga11G0898.1	*GhMEKK39*	Gorai.007G053300.1
GhMEKK18	Ga12G2908.1	*GhMEKK40*	Gorai.007G213900.1
GhMEKK19	Ga12G2339.1	*GhMEKK41*	Gorai.007G296700.1
GhMEKK20	Ga12G1545.1	*GhMEKK42*	Gorai.008G000500.1
GhMEKK21	Ga13G2820.1	*GhMEKK43*	Gorai.008G073600.1
GhRAF1	Ga01G1576.1	*GhMEKK44*	Gorai.008G077000.1
GhRAF2	Ga02G1149.1	*GhMEKK45*	Gorai.008G149400.1
GhRAF3	Ga03G0041.1	*GhMEKK46*	Gorai.013G269600.1
GhRAF4	Ga03G0070.1	*GhRAF46*	Gorai.002G002100.1
GhRAF5	Ga03G0390.1	*GhRAF47*	Gorai.002G159900.1
GhRAF6	Ga03G0610.1	*GhRAF48*	Gorai.002G201200.1
GhRAF7	Ga03G0611.1	*GhRAF49*	Gorai.005G004900.1
GhRAF8	Ga02G0049.1	*GhRAF50*	Gorai.005G007700.1
GhRAF9	Ga03G1413.1	*GhRAF51*	Gorai.005G044700.1
GhRAF10	Ga04G0943.1	*GhRAF52*	Gorai.005G067300.1
GhRAF11	Ga04G0244.1	*GhRAF53*	Gorai.005G142900.1
GhRAF12	Ga05G0062.1	*GhRAF54*	Gorai.003G005100.1
GhRAF13	Ga05G0456.1	*GhRAF55*	Gorai.001G261300.1
GhRAF14	Ga05G0706.1	*GhRAF56*	Gorai.012G064800.1
GhRAF15	Ga05G1937.1	*GhRAF57*	Gorai.009G408200.1
GhRAF16	Ga04G1468.1	*GhRAF58*	Gorai.012G157000.1
GhRAF17	Ga04G1511.1	*GhRAF59*	Gorai.009G045200.1
GhRAF18	Ga14G0424.1	*GhRAF60*	Gorai.009G190600.1
GhRAF19	Ga06G0496.1	*GhRAF61*	Gorai.010G033900.1

GhRAF20	Ga06G1869.1	*GhRAF62*	Gorai.010G185600.1
GhRAF21	Ga07G0011.1	*GhRAF63*	Gorai.001G001200.1
GhRAF22	Ga07G0240.1	*GhRAF64*	Gorai.001G022800.1
GhRAF23	Ga07G2547.1	*GhRAF66*	Gorai.001G266100.1
GhRAF24	Ga07G2599.1	*GhRAF67*	Gorai.004G077800.1
GhRAF25	Ga08G0757.1	*GhRAF68*	Gorai.004G101000.1
GhRAF26	Ga08G1003.1	*GhRAF69*	Gorai.006G034500.1
GhRAF27	Ga14G1817.1	*GhRAF70*	Gorai.006G056600.1
GhRAF28	Ga09G0686.1	*GhRAF71*	Gorai.006G080100.1
GhRAF29	Ga14G0576.1	*GhRAF72*	Gorai.006G233100.1
GhRAF30	Ga09G1563.1	*GhRAF73*	Gorai.006G234800.1
GhRAF31	Ga09G2426.1	*GhRAF74*	Gorai.006G241500.1
GhRAF32	Ga09G2438.1	*GhRAF75*	Gorai.006G258400.1
GhRAF33	Ga09G2365.1	*GhRAF76*	Gorai.011G010500.1
GhRAF34	Ga09G2670.1	*GhRAF77*	Gorai.011G131000.1
GhRAF35	Ga10G2994.1	*GhRAF78*	Gorai.011G167400.1
GhRAF36	Ga10G1766.1	*GhRAF79*	Gorai.011G200300.1
GhRAF38	Ga10G1015.1	*GhRAF81*	Gorai.007G076700.1
GhRAF39	Ga11G3372.1	*GhRAF82*	Gorai.007G139200.1
GhRAF40	Ga11G2193.1	*GhRAF83*	Gorai.007G190200.1
GhRAF41	Ga12G2886.1	*GhRAF84*	Gorai.008G003000.1
GhRAF42	Ga12G1203.1	*GhRAF85*	Gorai.008G179300.1
GhRAF43	Ga12G0091.1	*GhRAF86*	Gorai.008G294700.1
GhRAF44	Ga12G0039.1	*GhRAF87*	Gorai.013G089100.1
GhRAF45	Ga13G0934.1	*GhZIK13*	Gorai.002G096100.1
GhZIK1	Ga01G0928.1	*GhZIK14*	Gorai.005G042400.1
GhZIK2	Ga03G0365.1	*GhZIK15*	Gorai.012G033500.1
GhZIK3	Ga01G1804.1	*GhZIK16*	Gorai.001G064400.1
GhZIK4	Ga04G1748.1	*GhZIK17*	Gorai.004G150400.1
GhZIK5	Ga07G0665.1	*GhZIK18*	Gorai.006G269500.1
GhZIK6	Ga08G1482.1	*GhZIK19*	Gorai.011G128900.1
GhZIK7	Ga09G2783.1	*GhZIK20*	Gorai.003G069600.1
GhZIK8	Ga10G1803.1	*GhZIK21*	Gorai.007G285300.1
GhZIK9	Ga14G2590.1	*GhZIK22*	Gorai.008G257300.1
GhZIK10	Ga11G0996.1	*GhZIK23*	Gorai.013G156000.1
GhZIK11	Ga12G0406.1	*GhZIK24*	Gorai.013G272100.1
GhZIK12	Ga14G0657.1		

Table S3. Primers used in vector construction and quantitative RT-PCR analysis.

Gene	Primer
GhRAF4-F	GGAATTCCAAGGACAGCCATTCAGACCC
GhRAF4-R	GGGTACCCTGGACGTGCTAATTTGGCCT
GhMEKK12-F	GGAATTCCTGCCGTTAAGTCCTCTGAGC
GhMEKK12-R	GGGTACCCATGTCAGCTGGGAACCCTTG
GhMEKK10-RT-F	GCTTGGCGATCCACCAATCAGA
GhMEKK10-RT-R	CGCCGCCGAGGATTCATTCTTA
GhMEKK12-RT-F	GAAGAAGAAGCGTGGGTTAGCAG
GhMEKK12-RT-R	TGCCCACTAATCTCTAAGTCTAAGCC
GhMEKK24-RT-F	TTTCGTCTCGAAGGAACCGACG
GhMEKK24-RT-R	CCGAGGCAGAAGATCCGATGAC
GhMEKK31-RT-F	TTTCGTCTCGAAGGAACCGACG
GhMEKK31-RT-R	CCGAGGCAGAAGATCCGATGAC
GhMEKK36-RT-F	CGGATTCTCATCTGATGTGCCTGA
GhMEKK36-RT-R	CAATACGCTGCTGGGTGTCTCA
GhMEKK40-RT-F	ACGGTTGAGAAGGGTGAGAAGT
GhMEKK40-RT-F	GGATTCAATCAAGTTTCCGCCGC
GhRAF4-RT-F	CCCACCGAAGAGGCCGTAAAAT
GhRAF4-RT-R	CCAACATTCTCCGATGAGCTCCC
GhRAF8-RT-F	CTGGCAACAGGACCCAACTCAA
GhRAF8-RT-R	GGCGATCTTCCCCCTTATCTGC
GhRAF21-RT-F	GAACCGTGCTGTGCCATTGAAG
GhRAF21-RT-R	TCTGGTGTCATGCCCTCAGTCT
GhRAF78-RT-F	CTGCGAGCATCATTTCGGCAAG
GhRAF78-RT-R	CCATTGAGGCCCCCACAAACTT
GhRAF87-RT-F	TCTTACGGCCAAAGTGGAAGCC
GhRAF87-RT-R	GACAGCTACAGGTGTTCCTCGC
GhUBI1-F	CTGAATCTTCGCTTTCACGTTATC
GhUBI1-R	GGGATGCAAATCTTCGTGAAAAC

A

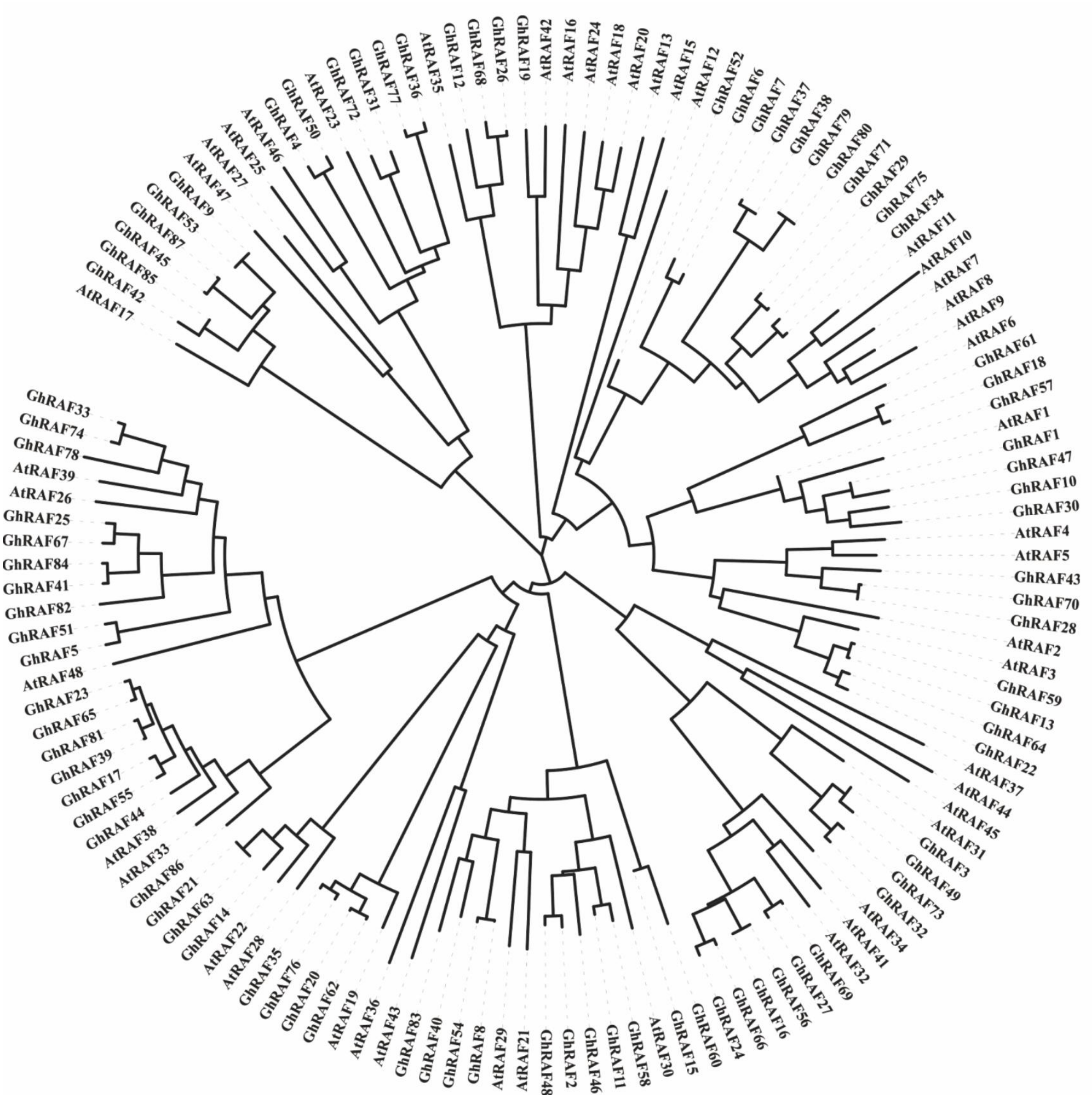
AtRAF17
GhRAF42
GhRAF85
GhRAF45
GhRAF87
GhRAF53
GhRAF9
AtRAF47
AtRAF27
AtRAF25
AtRAF46
GhRAF4
GhRAF50
AtRAF23
GhRAF72
GhRAF31
GhRAF77
GhRAF36
AtRAF35
GhRAF12
GhRAF68
GhRAF26
GhRAF19
AtRAF42
AtRAF16
AtRAF24
AtRAF18
AtRAF20
AtRAF13
AtRAF15
AtRAF12
GhRAF52
GhRAF6
GhRAF7
GhRAF37
GhRAF38
GhRAF79
GhRAF80
GhRAF71
GhRAF29
GhRAF75
GhRAF34
AtRAF11
AtRAF10
AtRAF7
AtRAF8
AtRAF9
AtRAF6
GhRAF61
GhRAF18
GhRAF57
AtRAF1
GhRAF1
GhRAF47
GhRAF10
GhRAF30
AtRAF4
AtRAF5
GhRAF43
GhRAF70
GhRAF28
AtRAF2
AtRAF3
GhRAF59
GhRAF13
GhRAF64
GhRAF22
AtRAF37
AtRAF44
AtRAF45
AtRAF31
GhRAF3
GhRAF49
GhRAF73
GhRAF32
AtRAF34
AtRAF41
AtRAF32
GhRAF69
GhRAF27
GhRAF56
GhRAF16
GhRAF66
GhRAF24
GhRAF60
GhRAF15
AtRAF30
GhRAF58
GhRAF11
GhRAF46
GhRAF2
GhRAF48
AtRAF21
AtRAF29
GhRAF8
GhRAF54
GhRAF40
GhRAF83
AtRAF43
AtRAF36
AtRAF19
GhRAF62
GhRAF20
GhRAF76
GhRAF35
AtRAF28
AtRAF22
GhRAF14
GhRAF63
GhRAF21
GhRAF86
AtRAF33
AtRAF38
GhRAF44
GhRAF55
GhRAF17
GhRAF39
GhRAF81
GhRAF65
GhRAF23
AtRAF48
GhRAF5
GhRAF51
GhRAF82
GhRAF41
GhRAF84
GhRAF67
GhRAF25
AtRAF26
AtRAF39
GhRAF78
GhRAF74
GhRAF33

B

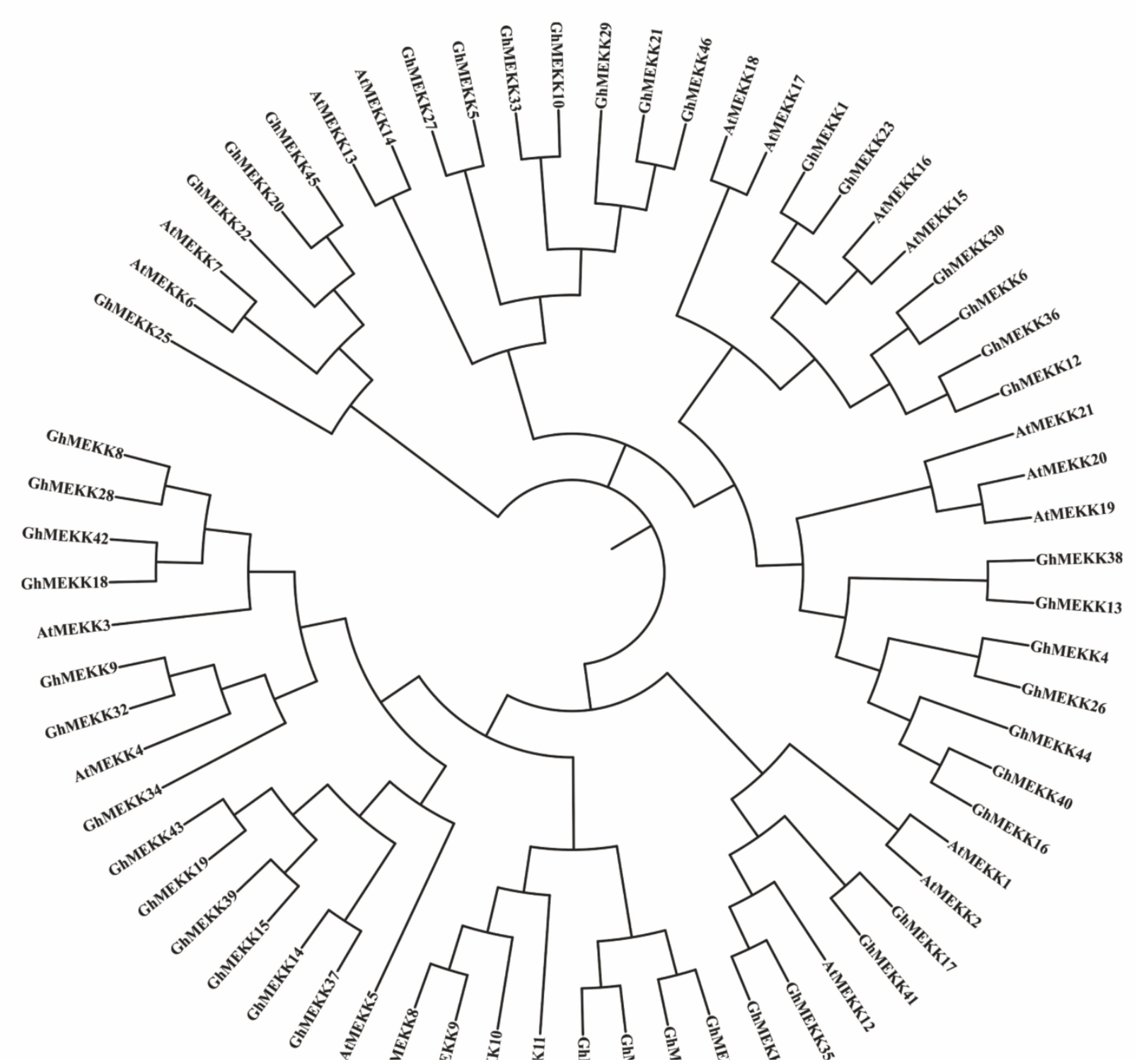
GhMEKK25
AtMEKK6
AtMEKK7
GhMEKK22
GhMEKK20
GhMEKK45
AtMEKK13
AtMEKK14
GhMEKK27
GhMEKK5
GhMEKK33
GhMEKK10
GhMEKK29
GhMEKK21
GhMEKK46
AtMEKK18
AtMEKK17
GhMEKK1
GhMEKK23
AtMEKK16
AtMEKK15
GhMEKK30
GhMEKK6
GhMEKK36
GhMEKK12
AtMEKK21
AtMEKK20
AtMEKK19
GhMEKK38
GhMEKK13
GhMEKK4
GhMEKK26
GhMEKK44
GhMEKK40
GhMEKK16
AtMEKK1
AtMEKK2
GhMEKK17
GhMEKK41
AtMEKK12
GhMEKK35
AtMEKK5
GhMEKK37
GhMEKK14
GhMEKK15
GhMEKK39
GhMEKK19
GhMEKK43
GhMEKK34
AtMEKK4
GhMEKK32
GhMEKK9
AtMEKK3
GhMEKK18
GhMEKK42
GhMEKK28
GhMEKK8

C

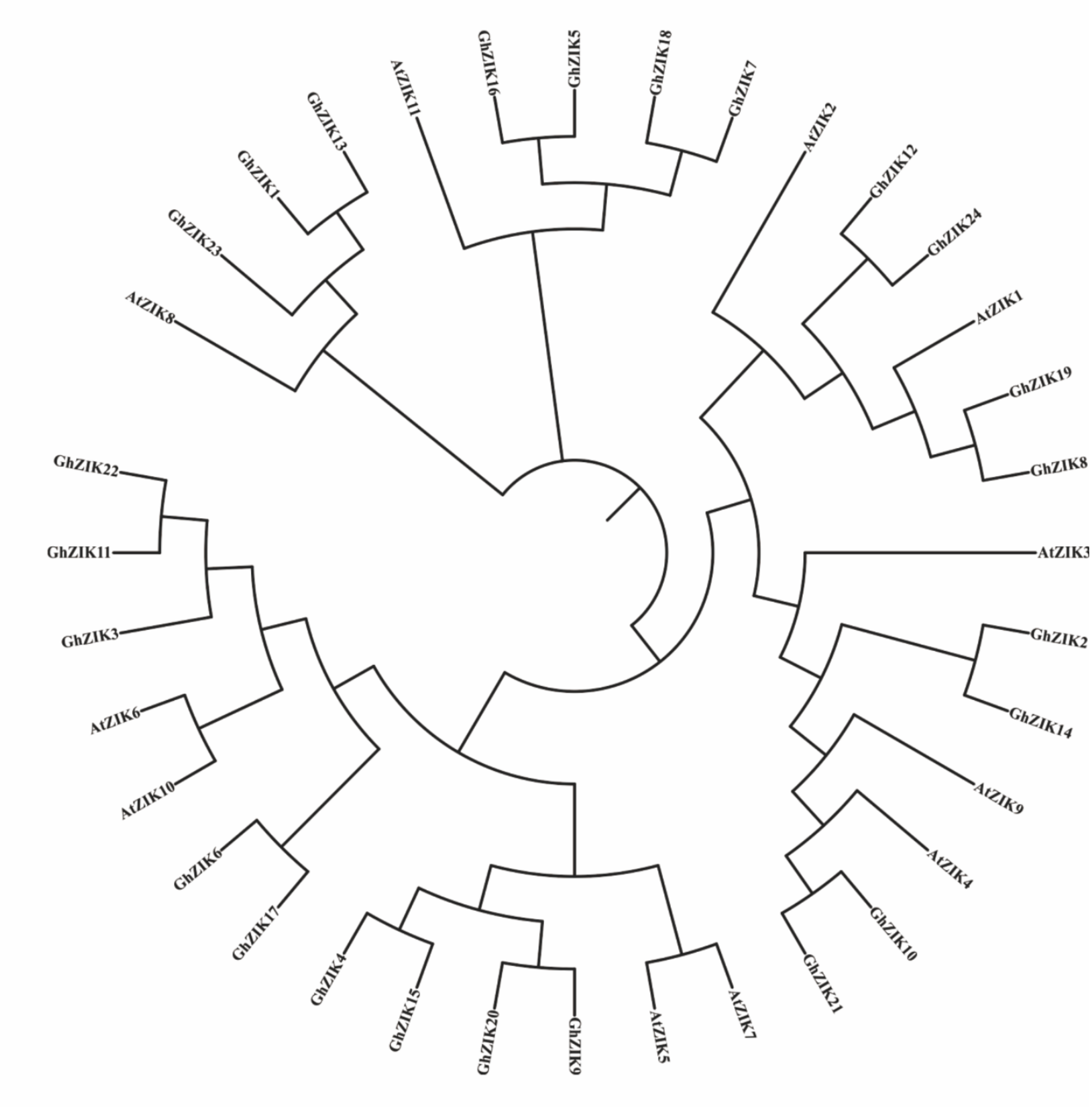

Figure S1. Phylogenetic relationship of GhMAPKKKs and AtMAPKKKs. (**A**) Phylogenetic relationship of GhRAFs and AtRAFs. (**B**) Phylogenetic relationship of GhRAFs and AtMEKKs. (**C**) Phylogenetic relationship of GhZIKs and AtZIKs. The unrooted phylogenetic tree was constructed using MEGA 6.0 by Neighbor-Joining method and the bootstrap test was performed with 1,000 iterations. Gh, upland cotton; At, Arabidopsis.

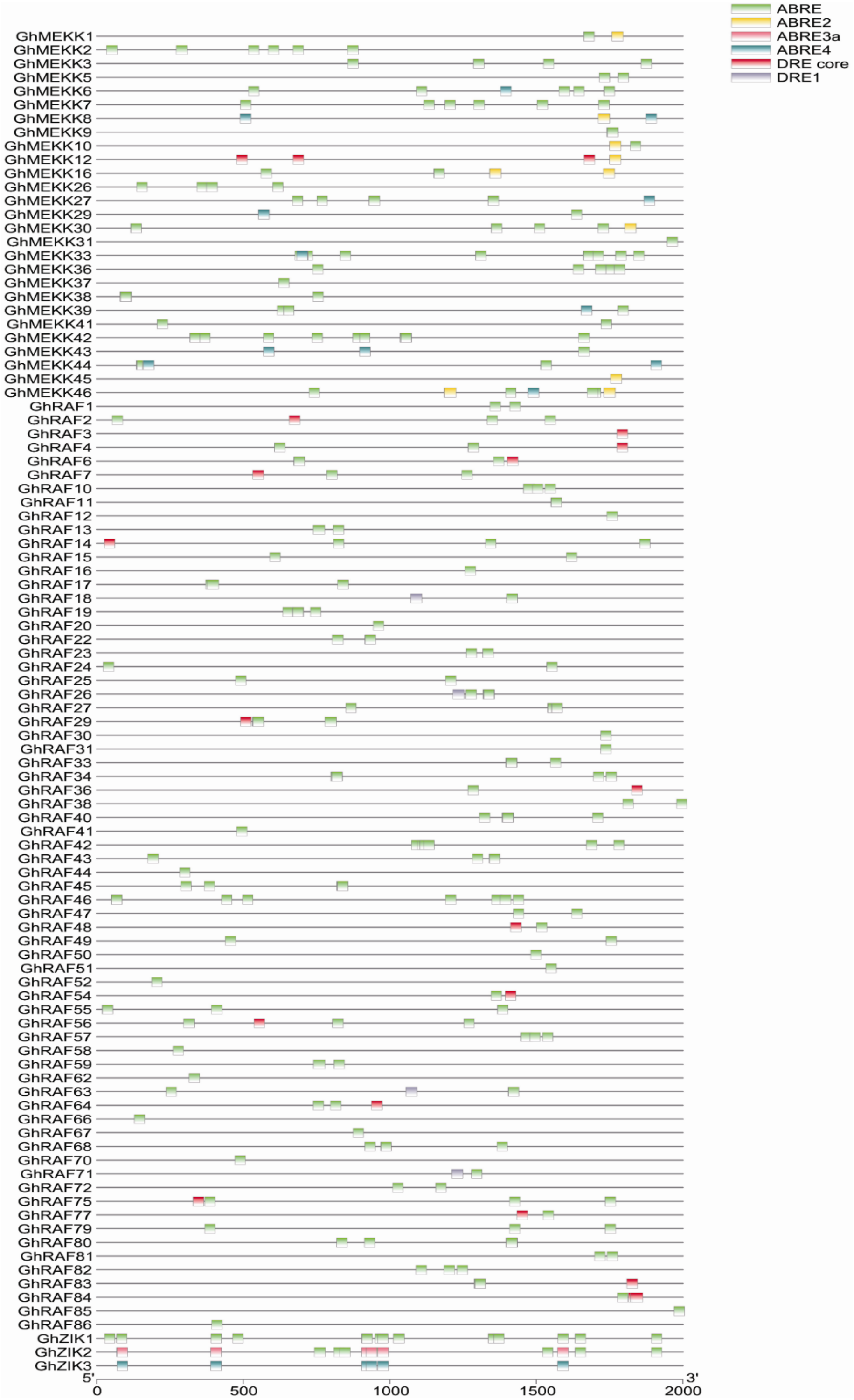

Figure S2. Analysis of *GhMAPKKK* promoters. The cis-elements existed in the promoter sequences were identified in cotton MAPKKK gene family. Each cis-element is indicated with a specific color.
